# Leaderless Bicistronic Design for Precise and Reliable Control of Gene Expression in *Corynebacterium Glutamicum*

**DOI:** 10.1021/acssynbio.3c00246

**Published:** 2023-06-23

**Authors:** Xiuxia Liu, Manman Sun, Alex Xiong Gao, Rodrigo Ledesma-Amaro, Qiuwu Fang, Yankun Yang, Zhonghu Bai

**Affiliations:** † National Engineering Research Center of Cereal Fermentation and Food Biomanufacturing, Jiangnan University, Wuxi 214112, China; ‡ Key Laboratory of Industrial Biotechnology, Ministry of Education, School of Biotechnology, Jiangnan University, Wuxi 214122, China; § Jiangsu Provincial Research Center for Bioactive Product Processing Technology, Jiangnan University, Wuxi 214122, China; ∥ Department of Bioengineering and Imperial College Centre for Synthetic Biology, Imperial College London, London SW7 2AZ, U.K.; ⊥ Division of Life Science, The Hong Kong University of Science and Technology, Hong Kong, China; # Living Systems Institute, School of Biosciences, College of Life and Environmental Science, University of Exeter, Exeter EX4 4QD, U.K.

**Keywords:** leaderless bicistronic design, improved orthogonality, gene expression regulation, Corynebacterium glutamicum, synthetic biology

## Abstract

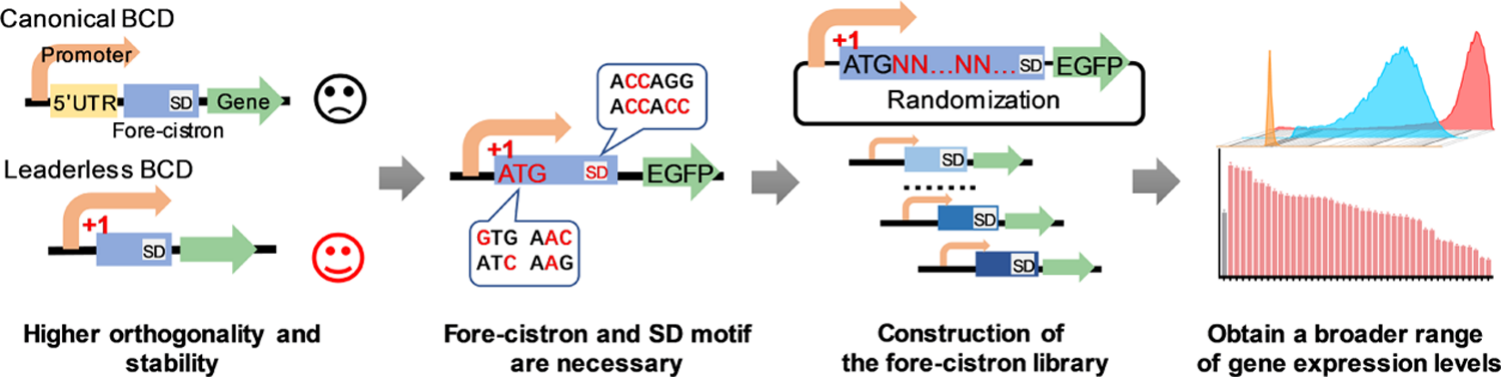

In synthetic biology, the precise control of gene expression is challenging due to the limited orthogonality of expression elements. Here, to address this issue and improve the reusability of genetic elements, we developed a bicistronic expression cassette in *Corynebacterium glutamicum* based on a leaderless promoter lacking a 5′UTR. The created leaderless bicistronic design (BCD) significantly improved the orthogonality of expression elements across different genes of interest. We also explored the importance of the fore-cistron and SD motif in maintaining the strength of leaderless BCDs. Additionally, we established a library containing 55,901 fore-cistrons and demonstrated that the regulatory range of gene expression in leaderless BCDs can be broader by modifying the fore-cistron sequence. This study provides a novel synthetic biology tool based on leaderless BCD for fine-tuning gene expression in *C. glutamicum* using fore-cistrons. Moreover, the strategy developed here can also be applied to improve the performance of other leaderless promoters in other bacteria.

## Introduction

The modulation of gene expression is a crucial aspect of synthetic biology and metabolic engineering,^1,2^ but achieving precise control over gene expression can be complex and involve multiple regulatory factors.^3−5^ Two key steps in defining gene expression are the interaction of the promoter with RNA polymerase and the binding of the ribosome to the ribosome-binding site (RBS), as they directly determine the initiation of transcription and translation.^6−8^ Therefore, promoters and RBSs are key regulatory elements for fine-tuning gene expression in synthetic biology. In recent decades, numerous endogenous promoters,^9,10^ promoter mutants,^11^ synthetic promoters,^12,13^ and RBS libraries^14,15^ have been developed, characterized, and used to vary gene expression. Researchers have also developed other regulatory elements, such as terminators, small regulatory RNAs, or proteolysis tags, which can be applied for gene expression control.^1^ Despite these extensive advances, the precise and reliable control of gene expression is still challenging, as these functional genetic parts cannot be reliably reused in novel expression cassettes.^16,17^

To overcome these challenges, much effort has been devoted to developing strategies and tools to reduce the variation in gene expression levels arising from different genetic contexts.^18−20^ A representative strategy is bicistronic expression.^21^ In 2013, Mutalik et al. demonstrated for the first time that a bicistronic design (BCD) could be used to reduce the impact of the irregular 5′-untranslated region (5′UTRs) and target gene junctions on gene expression levels.^22^ In their expression cassette, a short leader peptide coding sequence (fore-cistron) ended with a second RBS (SD2) inserted upstream of the target gene. Ribosomes recruited by the translation of fore-cistron can open up unfavorable mRNA secondary structures in the translation initiation region (TIR) of the downstream target gene, favoring its translation initiation.^21^ A recent study by Duan et al. in 2022 further proved that BCD structures can improve the orthogonality and reliability of RBS.^6^ However, previous studies on the design and application of BCD have focused on canonical promoters containing a 5′UTR. A potential problem with the presence of 5′UTR is that different 5′UTR-fore-cistron junctions will form when combined with different promoters. This can cause undesirable variation in gene expression levels, thereby reducing the reusability of expression elements in BCD.

Over the past decades, due to the advances in RNA sequencing technology, researchers have found that in bacteria, archaea, and extremophiles, some mRNAs are able to initiate translation at the transcription start point (TSP) and lack 5′UTR.^23−26^ This kind of transcript is called leaderless mRNA (lmRNA), and the promoter that controls lmRNA transcription is called a leaderless promoter here. Due to the absence of 5′UTR, the effect of 5′UTR on gene expression is eliminated.^24^ Therefore, we hypothesized that BCD expression cassettes based on leaderless promoters would more reliably control gene expression. In this study, we developed and explored the applicability of leaderless bicistronic promoters in *Corynebacterium glutamicum*, an important industrial bacterium for the production of amino acids, biochemicals, and recombinant proteins.^27−30^ This study also demonstrated the necessity of the translation of fore-cistron and an active Shine–Dalgarno (SD) motif in maintaining leaderless BCD expression intensity. Finally, a fore-cistron library was established, enabling a broader range of gene expression regulation.

## Results and Discussion

### Exploration of the Application of Bicistronic Strategy in the Leaderless Promoter

**Figure 1:**
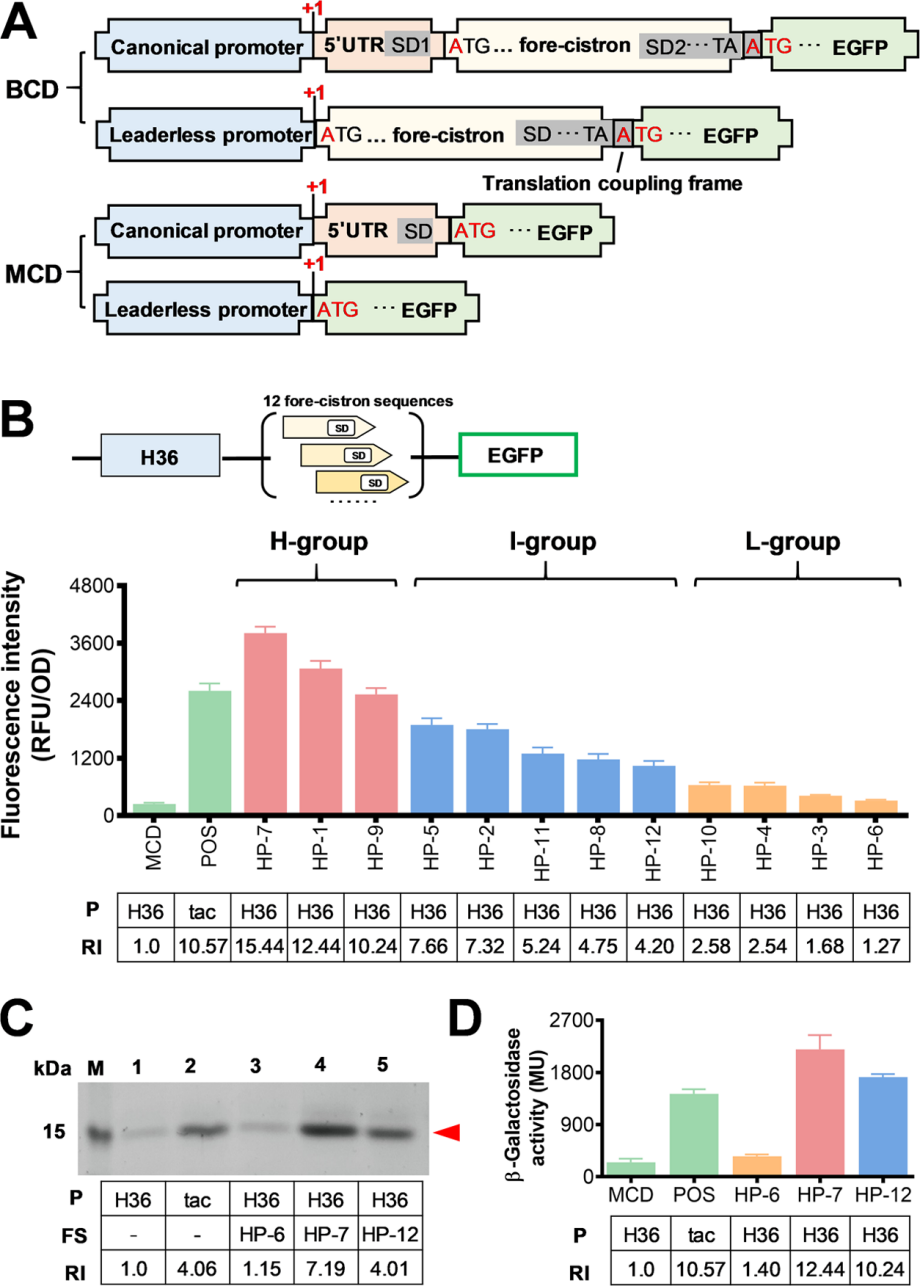
Expression structure and expression intensity of leaderless bicistronic expression cassettes. BCD: bicistronic design; MCD: monocistronic design; P: promoter; FS: fore-cistron sequence; RI: relative intensity. (A) Bicistronic and monocistronic expression structure of leaderless promoter. (B) Expression strength analysis of bicistronic P_H36_ as presented by the fluorescence intensities. MCD represents the EGFP fluorescence under the control of the monocistronic P_H36_, and HP-1 to HP-12 represent leaderless BCDs containing different fore-cistron sequences. pXMJ19-EGFP containing a monocistronic P_tac_ was used as the positive control (POS), and the error bars represent the standard deviation of biological triplicate. H, I, and L represent the high, intermediate, and low groups, respectively. (C) SDS-PAGE analysis of VHH expression in the leaderless BCDs. Lane M: protein marker; Lane 1–5: VHH production in different expression cassettes. (D) Activities of β-galactosidase in different expression cassettes.

Over the past decades, the BCD expression cassette has been applied in many prokaryotic systems, including *Escherichia coli*, *Corynebacterium glutamicum*, *Bacillus subtilis*, *Streptomyces* spp., etc.^21^ To evaluate whether the bicistronic strategy can be applied to the leaderless promoter in *C. glutamicum*, the previously developed synthetic promoter P_H36_^13^ was constructed in a bicistronic design (BCD) manner. The N-terminal 62 bp from the top 12 highly expressed genes in *C. glutamicum* (Table 1) were introduced as a fore-cistron sequence to ensure that the designed leaderless BCD exhibited strong gene expression performance,^31−33^ as gene sequences with higher protein expression abundance may possess greater translation efficiency than others. This leaderless bicistronic expression cassette is shown in Figure 1A. Each fore-cistron sequence includes a conserved SD sequence (AAAGGAGGACAAC) with a strong affinity for *C. glutamicum* 16S rRNA,^34^ which was used to initiate the translation of the downstream reporter gene EGFP. The stop codon (TAA) in the terminus of the SD motif overlaps 1 bp with the start codon (ATG) of the EGFP to form the widely used translation coupling frame TAATG.^21^ Moreover, to ensure the consistency of TSP, the start codons of all of these 12 fore-cistron sequences were unified into AUG (Table 1). The monocistronic expression plasmid pXMJ19-EGFP with a constitutive P_tac_ promoter was used as the positive control (POS). A 5′RACE experiment proved that all transcripts of these BCDs were leaderless (Table S3), and fluorescence results showed that a wide range of EGFP expression levels from 313 to 3,813 fluorescence intensity values was obtained in the 12 BCDs of P_H36_, which were 1.27–15.44 times that of the monocistronic P_H36_ (Figure 1B). In a conventional BCD expression cassette containing a 5′UTR, the helicase activity of the ribosome recruited by the first SD (SD1) can open up possible secondary structures around the target gene, thus minimizing the effect of gene context on the gene expression level.^21,22^ To assess the stability of the expression elements with different target gene sequences, these 12 BCD expression cassettes were categorized into three groups: high (H), intermediate (I), and low (L) strength based on the EGFP fluorescent intensity (Figure 1B). We then selected a representative BCD from each group and further validated their expression strength using two additional proteins: the variable domain of heavy chain of heavy-chain antibody (VHH)^35^ and LacZ.

**Table 1:** Sequence Information of 12 Fore-Cistrons

number	gene ID	gene name	sequence (5′-3′)^a^
HP-1	NCgl0572	groES	atggcaaacgtcaacatcaagccgcttgaggacaagatcctcgttcagatcaacgaagcaga
HP-2	NCgl2287	ndk	atgactgaacgtactctcatccttatcaagccagacggtgttaccaacggacacgtcggcga
HP-3	NCgl2673	NCgl2673	atgcctatcgcaactcccgaggtctataacgagatgctcgatcgtgctaaggaaggcggatt
HP-4	NCgl1041	tpx	atggggtccatggctaaaacacattttcaaggcaacgaaactgctacctccggcgaactgcc
HP-5	NCgl1316	NCgl1316	atgagcgagaattacagcaagattgtcgttggcactgatggatctaagtcgtcccttctagc
HP-6	NCgl2501	NCgl2501	atggcccgtgtagttgtcaatgtcatgcctaaggctgagattctggatccccaggggcaggc
HP-7	NCgl0390	gpmA	atgactaacggaaaattgattcttcttcgtcacggtcagagcgaatggaacgcatccaacca
HP-8	NCgl0935	eno	atggctgaaatcatgcacgtattcgctcgcgaaattctcgactcccgcggtaacccaaccgt
HP-9	NCgl2328	clpP	atgagcgatattcgtatggcagcccagggtgggcctggtttcggaaatgacgtctttgatcg
HP-10	NCgl0533	adk	atgcgactcgtactcctcggacctcccggtgctggtaagggcacccaggctgcaattctctc
HP-11	NCgl2473	NCgl2473	atgattggagcaccacccgacatgggcaatgtgtacaacaacatcaccgaaaccatcggcca
HP-12	NCgl2826	NCgl2826	atggctgtatacgaactcccagaactcgactacgcatacgacgctctcgagccacacatcgc

a Red base represents mutation.

As shown in Figure 1C,D, all three BCDs exhibited enhanced expression levels of VHH and LacZ, although the specific relative intensity of protein expression was different between the vectors. This could be due to differences in translation efficiency, mRNA stability, or protein stability arising from the different target gene sequences.^36,37^ Despite the differences in protein expression levels, the ranking of expression intensity for the three BCDs remained consistent across all three proteins, with HP-7 showing the highest expression intensity, followed by HP-12 and then HP-6. These results demonstrate that the use of a bicistronic strategy with a leaderless promoter exhibits excellent stability toward different target gene sequences, as we previously observed in conventional BCDs.^33^

### Evaluation of the Orthogonality of Genetic Elements in Leaderless BCDs

**Figure 2:**
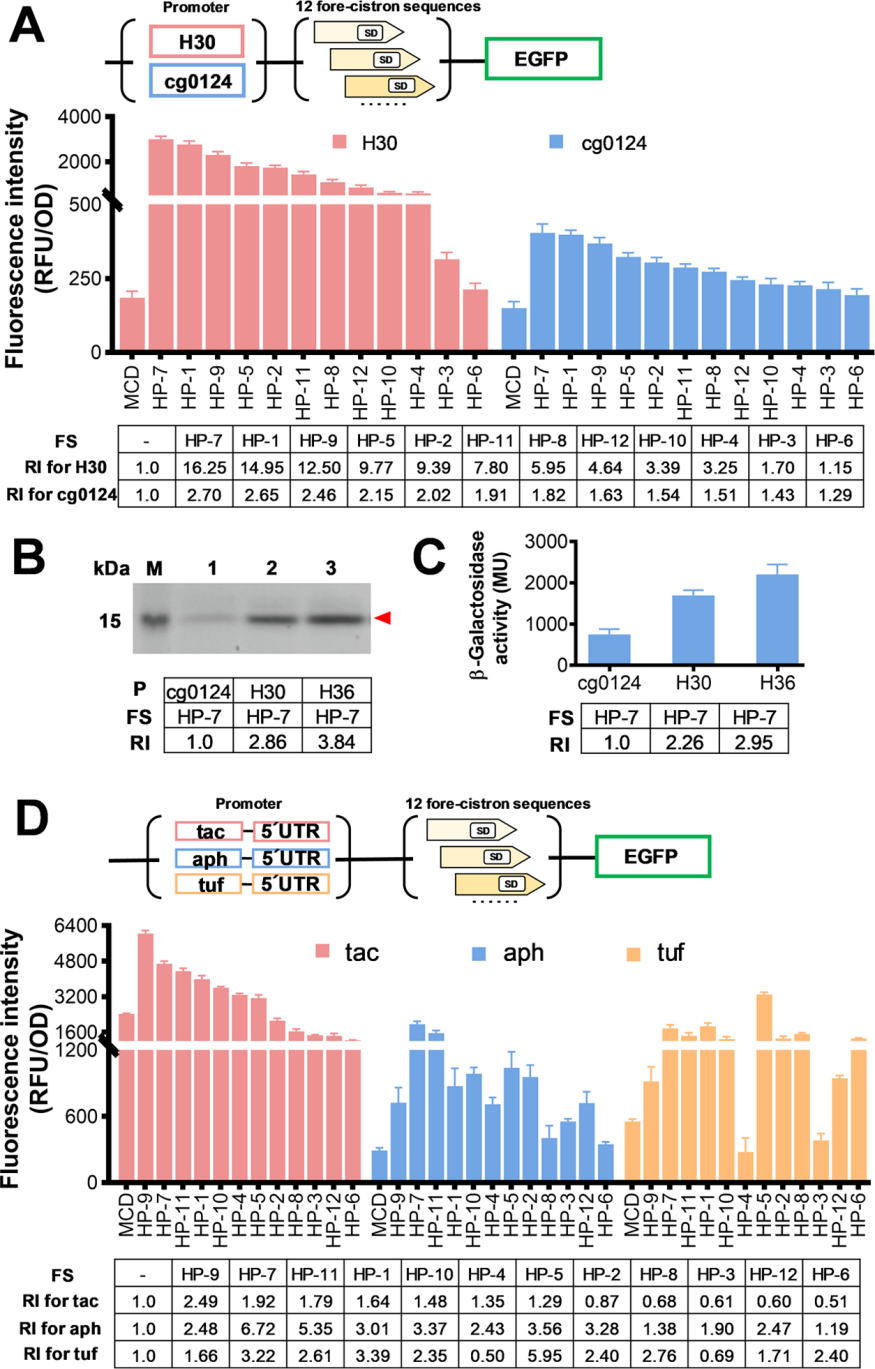
Evaluation of expression intensity of different bicistronic promoters. P: promoter; FS: fore-cistron sequence; RI: relative intensity. (A) Expression strength analysis of leaderless BCD as presented by the fluorescence intensities. MCD represents the EGFP fluorescence under the control of the corresponding monocistronic promoter, and HP-1 to HP-12 represent leaderless BCDs containing different fore-cistron sequences. For the RI analysis, the expression intensity of the corresponding MCD was defined as 1. (B) SDS-PAGE analysis of VHH expression under the control of different leaderless promoters. Lane M: protein marker; Lanes 1–3: VHH production under the control of different leaderless promoters. (C) Activities of β-galactosidase are under the control of different leaderless promoters. (D) Expression strength analysis of bicistronic expression cassettes containing a 5′UTR as presented by the fluorescence intensities. The expression intensity of the corresponding MCD was defined as 1.

**Figure 3:**
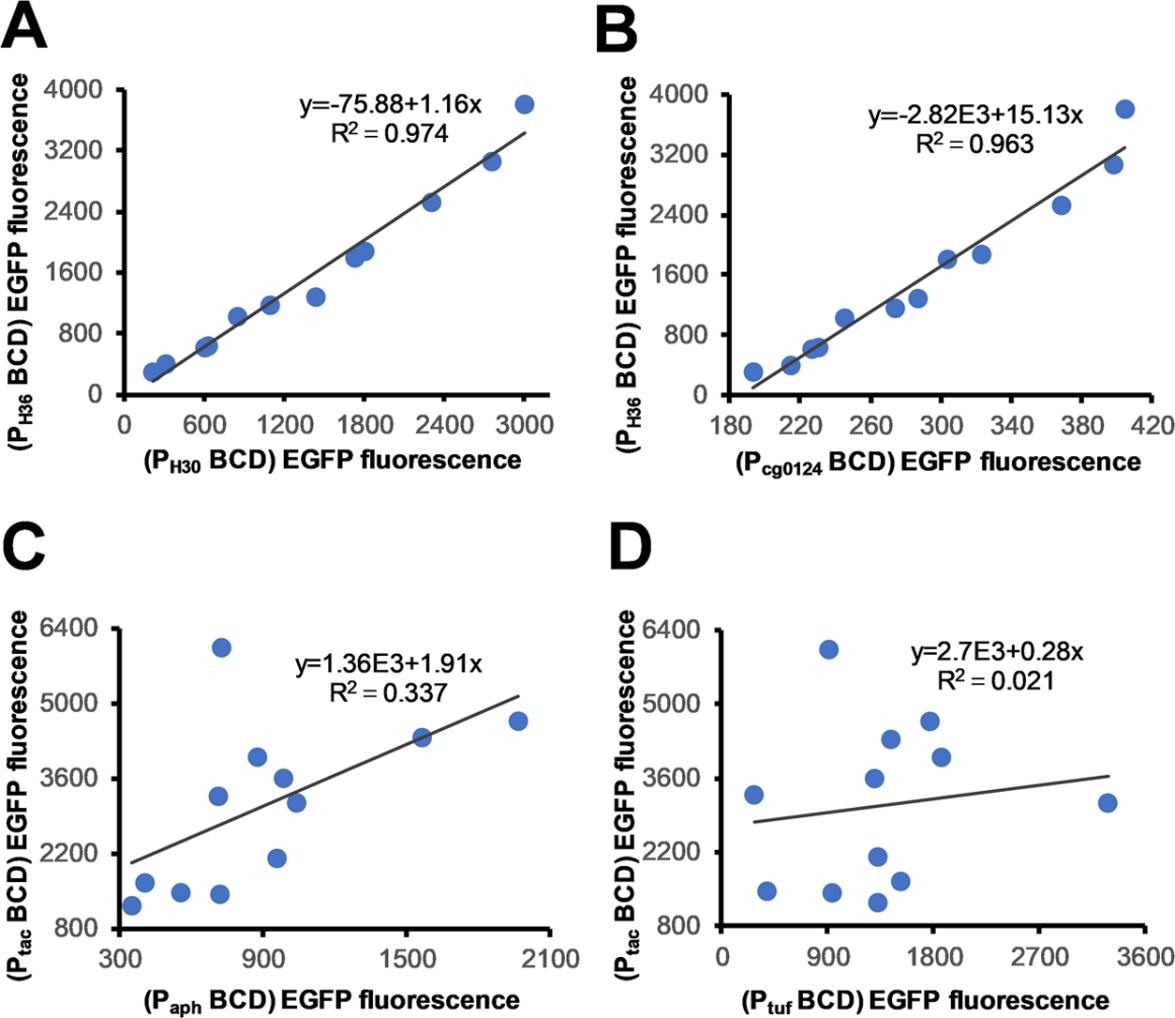
Correlation analysis of EGFP expression from BCDs regulated by different promoters. (A) Correlated gene expression levels from BCDs with a leaderless promoter P_H36_ or P_H30_. (B) Correlated gene expression levels from BCDs with a leaderless promoter P_H36_ or P_cg0124_. (C) Correlated gene expression levels from BCDs with promoter P_tac_ or P_aph_. (D) Correlated gene expression levels from BCDs with promoter P_tac_ or P_tuf_.

Transcription and translation are two key steps in gene expression. The transcriptional level is mainly dependent on promoter strength, while the translation efficiency is often strongly influenced by the 5′UTR.^24,38,39^ Due to the absence of the 5′UTR sequence in the leaderless BCDs, we hypothesize that gene expression is more orthogonal when combined with different leaderless promoters. To test our conjecture, another fully synthetic leaderless promoter P_H30_^40^ and an endogenous leaderless promoter P_cg0124_ were selected to construct BCD expression cassettes. The endogenous leaderless P_cg0124_ was selected based on the transcriptome data^41^ and 5′RACE experiment. The promoter sequence was determined using BDGP Neural Network Promoter Prediction.^31^Figure 2A shows EGFP fluorescence intensities of the generated 24 leaderless BCD expression cassettes (each promoter combined with 12 fore-cistrons). In the two leaderless promoters tested, all BCDs showed enhanced expression levels than the monocistronic design (MCD). Furthermore, these 12 BCDs containing different fore-cistron sequences showed a trend of strength consistent with that in P_H36_, and the activities of leaderless BCD in P_H30_ and P_cg0124_ remained well correlated with that obtained in P_H36_ (with a correlation coefficient of determination *R*^2^ = 0.974 and 0.963, respectively) (Figure 3A,B). The stability of these expression cassettes against different target genes was proved in the expression of VHH and LacZ (Figure 2B,C). Next, we tested the orthogonality of canonical BCD expression cassettes containing a 5′UTR using three promoters, P_tac_, P_aph_,^42^ and P_tuf_^43^ (Figure 1A). As shown in Figure 2D, in contrast to the overall improvement in expression with the leaderless promoters, some BCDs showed reduced expression levels in the presence of 5′UTR. Inconsistent trends and poor correlation in the expression intensities were also observed in these three promoters (Figures 2D and 3C,D). These results demonstrate the weaker orthogonality of genetic elements in canonical BCD. In contrast, leaderless BCD can eliminate the interference of the 5′UTR, providing higher orthogonality and stability. Therefore, leaderless BCD is a more reliable tool in synthetic biology for precise control of gene expression.

### SD Motif and the Fore-Cistron Translation Are Necessary for Maintaining the Strength of Leaderless BCDs

**Figure 4:**
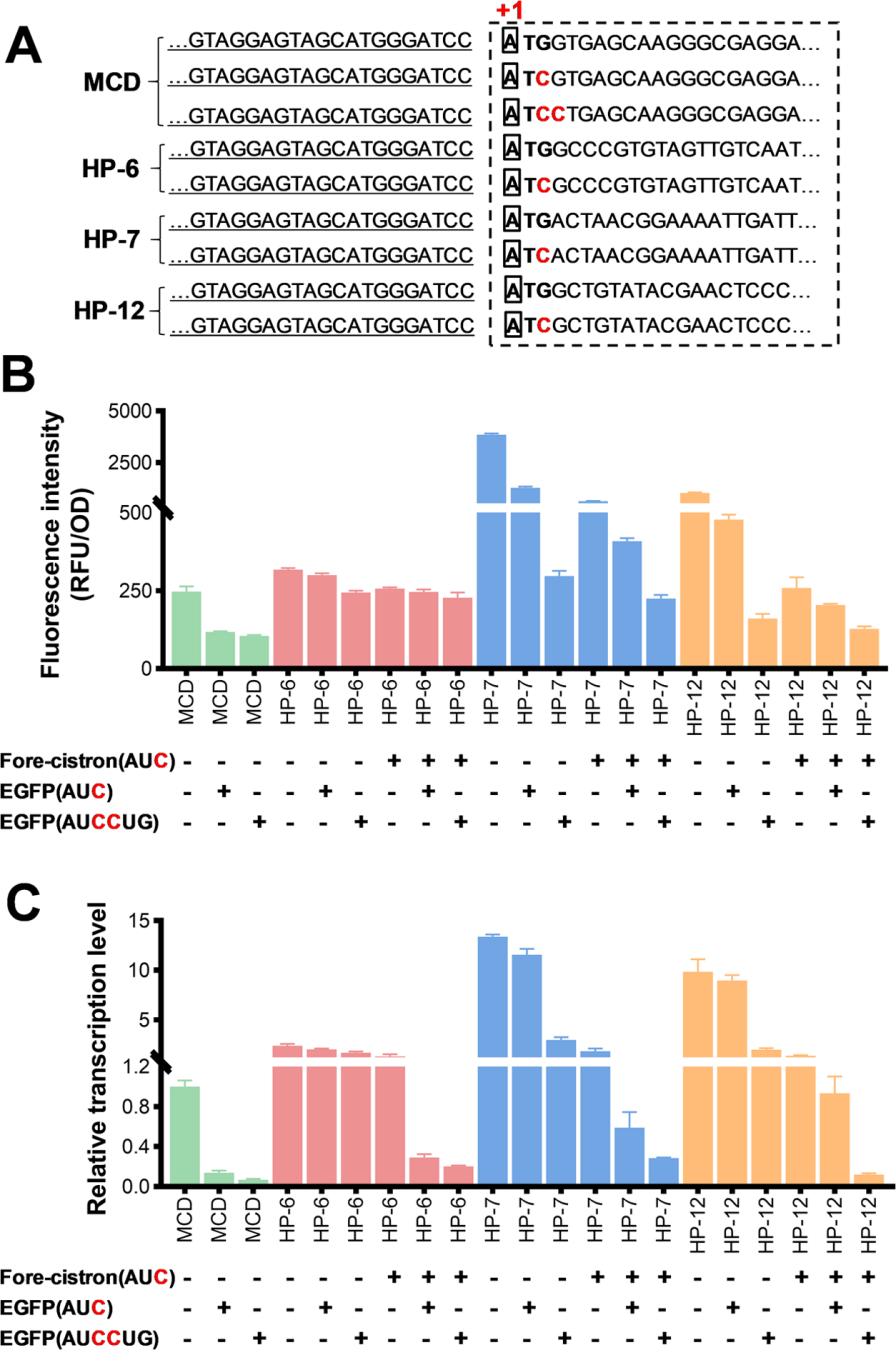
Effect of start codon mutation on the expression intensity of leaderless BCD. (A) Identification of transcription initiation sites (TSPs). 5′RACE-determined TSPs (+1) are given in bold letters with black boxes. The first codon triplet is shown in boldface, and the mutated base is shown in red characters. The underlined sequence represents the sequence of P_H36_ and the dashed boxes indicate the sequence of EGFP (MCD) or fore-cistron. (B) Effects of fore-cistron and EGFP start codon mutations on the expression intensity of leaderless BCD. The “+” represents a mutation of the start codon, and the mutated base is shown in red characters. The “–” indicates that the start codon is not mutated. (C) Effects of fore-cistron and EGFP start codon mutations on the transcriptional level of EGFP in leaderless BCD.

qRT-PCR results revealed that compared to MCD, the transcription levels of EGFP in all BCD expression cassettes were enhanced (Figure S1). To investigate the impact of the translation of the fore-cistron on the expression strength of leaderless BCDs, we mutated the start codon AUG of the fore-cistron into AUC (AUG → AUC). Furthermore, we mutated the initial codon of the downstream reporter gene EGFP into AUC (AUG → AUC) to evaluate the role of the fore-cistron when the target gene is not translated. Additionally, we mutated the second codon GUG of EGFP into CUG to prevent any possible translation initiation (AUGGUG → AUCCUG). The 5′RACE experiment confirmed that the transcripts of all constructs have the same TSP (Figure 4A), eliminating the impact of TSP inconsistency on the fluorescence results. As shown in Figure 4B,C, the mutation of the fore-cistron start codon in all constructed leaderless BCDs resulted in a significant reduction in both EGFP fluorescence and transcriptional level. This clearly indicates that the translation of the fore-cistron is essential for maintaining the high intensity of leaderless BCDs. Interestingly, we observed that the EGFP start codon mutation alone also caused decreased EGFP transcriptional level, which may be due to increased mRNA degradation caused by reduced translating ribosomes.^39,44^ Compared to BCDs with HP-12 and HP-7, the EGFP start codon mutation in the BCD with HP-7 resulted in a smaller reduction in EGFP fluorescence and transcription. This may be because the HP-6 is the weakest of the three, and further weakening on a weak basis may not result in significant differences as the stronger ones. Additionally, the pre-translated fore-cistron can deliver ribosome or 30S subunit to the downstream EGFP gene,^21^ which will further dilute the difference caused by the EGFP start codon mutation.

To further investigate the role of the fore-cistron initiation codon, we mutated it into AAC and AAG (AUG → AAC, AUG → AAG,). All of these mutated constructs started transcription at the same point and showed a significant reduction in EGFP fluorescence and transcription level (Figure S2). Notably, all leaderless BCDs with the fore-cistron mutated in the initiation codon (AUG → AUC) and either with wild-type or mutant EGFP (AUG → AUC) still exhibited higher EGFP fluorescence and transcriptional levels than their corresponding MCDs (Figure 4B,C). However, the observed improvements disappeared when the SD sequence was mutated (AGGAGG → ACCAGG, AGGAGG → ACCACC) (Figure S3). Taken together, these results suggest that an active SD motif and the translation of the fore-cistron are necessary to maintain the enhanced expression observed in leaderless BCDs.

### Regulatory Range of Gene Expression in Leaderless BCDs Can Be Broader by Modifying the Fore-Cistron

**Figure 5:**
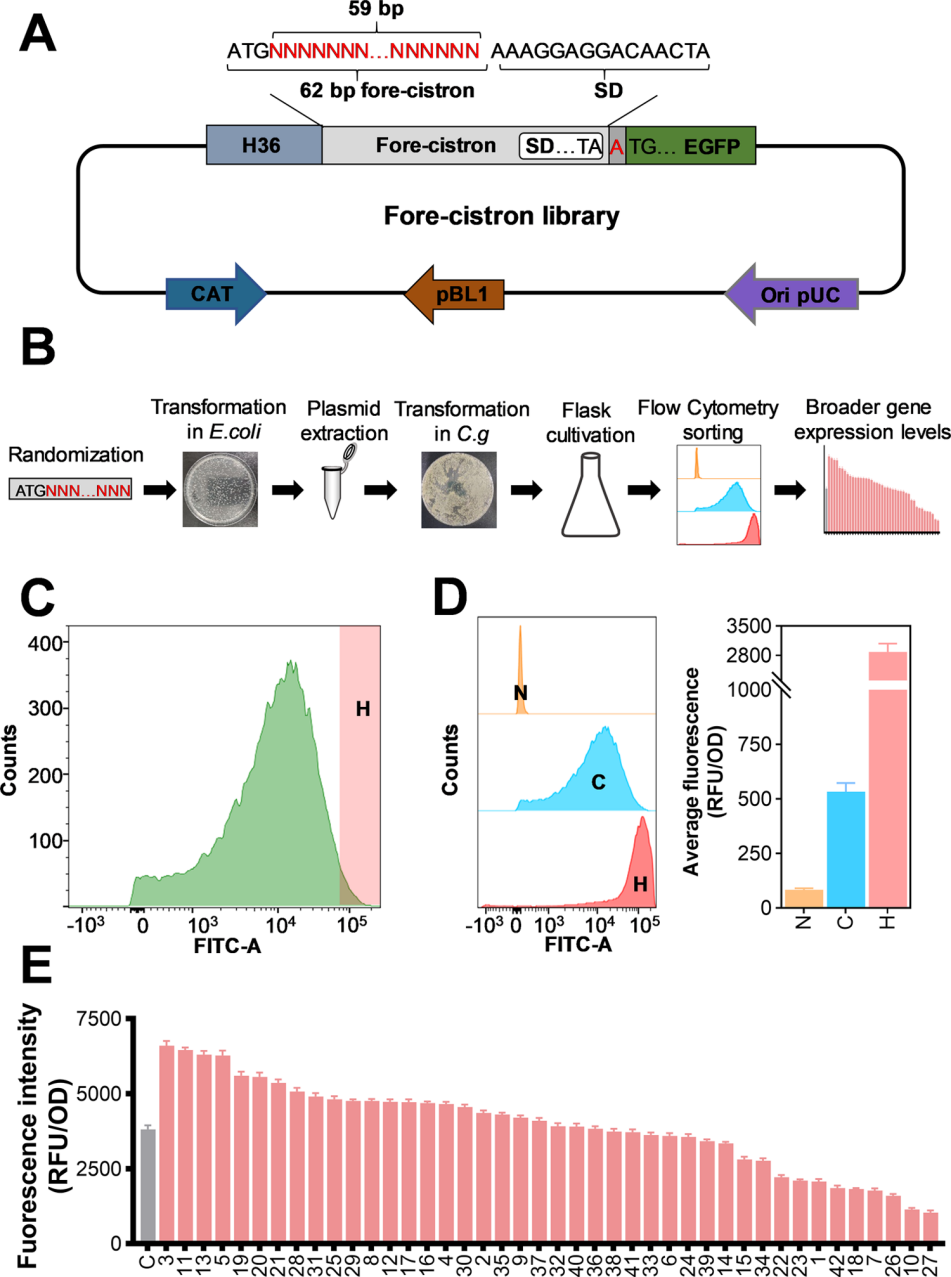
Construction and screening of the fore-cistron library. (A) Schematic diagram of plasmids used for fore-cistron library construction. The 62 bp fore-cistron sequence was fully designed with random bases (indicated with N) except for the start codon ATG (AUG). CAT: chloramphenicol resistance gene. (B) Workflow for the construction and screening of the fore-cistron library. (C) Flow cytometry screening of the fore-cistron library. H represents the high fluorescence group. (D) Average fluorescence intensity and the flow cytometry analysis. The wild-type *C. glutamicum* CGMCC1.15647 without EGFP expression was used as the negative control (N). The original library was used as the control (C). (E) EGFP fluorescence intensities of the isolated *C. glutamicum* clones randomly selected from the high (H) group. The control (C) represents the fluorescence intensity of H36-HP-7.

Previous studies have demonstrated that gene expression in BCD can be fine-tuned by engineering the SD sequence.^22,45^ As demonstrated above, in addition to the SD sequence, the fore-cistron also affects the expression of the gene of interest (GOI). However, there are currently no reports of engineering a library of fore-cistrons to fine-tune the expression of a downstream GOI. To expand the range of gene expression regulation, a library of 59 bp sequences downstream of the fore-cistron start codon ATG was designed to have fully random sequences, culminating in a conserved SD motif (Figure 5A). The fore-cistron library was initially introduced into *E. coli* JM109 to obtain a high number of transformants (ca. 5×10^5^ clones) and then transformed into *C. glutamicum* (around 5 × 10^5^ transformants) (Figure 5B). The diversity of the library was analyzed by NGS sequencing, and a total of 55,901 different fore-cistron sequences were identified (Table S5). Through flow cytometry sorting of cells displaying EGFP expression within the top 5%, a high sub-library (H-group) was generated (Figure 5C). As shown in Figure 5D, significant differences were observed in the fluorescence level of cells in the H-group and the original library. The high sub-library was further analyzed by randomly picking 42 clones; the fore-cistron sequences of these 42 clones are shown in Table S4. Among these 42 clones, 24 clones showed improved fluorescence intensity compared to the highest fluorescence previously observed with the H36 promoter (Figure 5E), further validating the reliability of the cell sorting results. Taken together, these results demonstrate that the fore-cistron sequence can act as a “regulator” with a broader range of gene expression levels in leaderless BCD.

## Conclusion and Comparison to Previous Studies

Benefiting from reduced mRNA secondary structure and efficient translation initiation, BCD expression cassettes typically exhibit higher expression levels than MCD.^21,33^ Previous studies have highlighted the importance of a well-translated fore-cistron^46,47^ and an active SD motif^48−50^ in maintaining a high expression strength in the traditional BCD containing a 5′UTR. Here, we have confirmed a similar observation in the leaderless BCD, where both fore-cistron start codon mutation and SD mutation resulted in reduced expression intensity (Figures 4, S2, and S3). In natural leaderless structures, the 5′-AUG has been observed to play an important role in the initial ribosome interaction with lmRNAs,^24,51^ which we also validated in the artificial leaderless BCD. However, the degree of reduction in EGFP fluorescence and transcription level observed from the AUG to GUG mutation (AUG → GUG) was lower compared to other start codon mutations (Figure S2). This suggests that although 5′-AUG is highly preferred, it is not strictly required for translation initiation in *C. glutamicum* leaderless transcripts, which has been previously observed in mycobacteria.^24^

Previous studies have highlighted the potential of BCDs in the precise control of gene expression.^6,22^ In this study, we further explored the application of this BCD strategy in leaderless promoters in *C. glutamicum*. Compared to the conventional BCD expression cassette with a 5′UTR, leaderless BCD has several advantages. First, the effect of the 5′UTR on gene expression is eliminated, improving the genetic elements’ orthogonality to the gene background and making the leaderless BCD structure more suitable for reliable gene expression control. Second, the lack of 5′UTR in the leaderless BCD simplifies the contents of the expression cassette, reducing the sequence space required for genetic modifications. Furthermore, leaderless BCDs offer more controllable genetic elements, such as fore-cistron and SD, compared to traditional monocistronic designs. Therefore, a broader range of gene expression regulation can be achieved by individually or simultaneously tuning these expression elements.

In conclusion, we extended the application of the BCD strategy in leaderless promoters and demonstrated their improved orthogonality when facing different genes of interest. The developed leaderless BCD could be an ideal tool for fine-tuning gene expression in *C. glutamicum* and holds promise for application in other bacterial systems as well. In addition, our findings showed that leaderless promoters typically exhibit weak expression strength when constructed in a traditional monocistronic manner (promoter–target gene), which may be unfavorable to studying the translation initiation mechanism of leaderless transcripts. Therefore, the leaderless BCD structure with higher expression strength developed in this study can also serve as an “amplifier” of leaderless promoters, facilitating the study of the translation initiation mechanism for leaderless transcripts.

## Materials and Methods

### Strains, Plasmids, and Cultural Conditions

The bacterial strains and plasmids used in this study are listed in Table S1. Plasmid construction was performed in *Escherichia coli* JM109. *C. glutamicum* CGMCC1.15647 was used as the host for protein expression, and the genome of *C. glutamicum* ATCC13032 provided the template for amplifying the endogenous promoters P_cg0124_ and P_tuf_. *E. coli* JM109 was cultivated at 37 °C with shaking at 220 rpm in LB medium or on LB plates with 2% (w/v) agar. *C. glutamicum* strains were transformed using LBHIS medium (5 g tryptone, 5 g NaCl, 2.5 g yeast extract, 91 g sorbitol, 18.5 g brain heart infusion per liter, pH 7.0). Unless otherwise indicated, *C. glutamicum* strains were cultivated in LBB medium (LB broth with 10 g brain heart infusion per liter) at 30 °C with shaking at 220 rpm. Chloramphenicol was added at a final concentration of 10 mg/L for *C. glutamicum* and 30 mg/L for *E. coli*. For inducible expression, Isopropyl β-d-Tiogalactoside (IPTG) was added at a final concentration of 1 mM when the OD_600_ of *C. glutamicum* cells reached about 1.0.

### Plasmid Construction

All primers used are listed in Table S2, and the plasmid P_btac_-HP-1 was used as a skeleton for plasmid construction.^33^ To form different bicistronic P_H36_ expression cassettes, the 95 bp leaderless promoter P_H36_ and the 62 bp fore-cistron sequence, obtained by primer annealing, were inserted into the linearized P_btac_-HP-1 (*Eco*RV/*Hin*dIII) through homologous recombination. For the monocistronic expression cassette, the P_H36_ fragment was directly ligated to the *Eco*RV/*Hin*dIII-digested P_btac_-HP-1. In all constructed plasmids, the *Hin*dIII restriction enzyme site was removed. The expression cassette for the other two leaderless promoters (P_H30_ and P_cg0124_), start codon mutants, SD mutants, and three traditional promoters containing a 5′UTR (P_tac_, P_aph_, and P_tuf_) were constructed in the same way as above. All DNA manipulation, including PCR, sequence digestion, ligation, Gibson assembly, etc., were performed according to the standard procedures of the instructions. The plasmids successfully constructed in *E. coli* JM109 were finally transformed into *C. glutamicum* CGMCC1.15647 by electroporation.

### 5′RACE Assay

5′rapid amplification of cDNA ends (5′-RACE) was used to identify the TSP of the promoter. *C. glutamicum* cells in the logarithmic growth phase were collected, and total RNA was extracted using the total RNA extraction kit from TaKaRa. The first strand of cDNA was reverse-transcribed using the gene-specific primer (GSP1) and the reverse transcription kit from Tsingke Biotechnology (TSK302M). After reverse transcription, the cDNA was purified using a DNA purification kit. Then, the dCTP was continuously added to the 3′ end of the cDNA strand by a terminal transferase (TaKaRa, D2230) to form an oligonucleotide poly C tail. Finally, a nested PCR amplification was performed using an oligo anchor primer (AP) and another gene-specific primer (GSP2) within the gene fragment. The PCR product was purified and ligated to the T-vector for sequencing to determine the TSP.

### Fluorescence Intensity Assay

EGFP was used as a reporter to assess the expression intensities of different BCD vectors. Cells harboring EGFP plasmids were cultivated overnight in a 24-deep-well plate containing 2 mL of LBB medium per well. Then, 200 μL cultures were transferred to 1.8 mL of fresh medium and grown for 24 h. The fluorescence intensity was measured by a fluorescence spectrophotometer as described previously.^33^ The fluorescence intensity per OD_600_ was calculated to indicate the expression intensity of different plasmids.

### Quantitative Reverse Transcription-PCR (qRT-PCR)

To analyze the transcription level of EGFP in different plasmids, *C. glutamicum* cells were cultivated in an LBB medium as described above. 1 mL of the culture was collected by centrifugation and washed with precooled PBS. Total RNA extraction, cDNA synthesis, and RT-PCR were performed using corresponding kits from TaKaRa. The RT-PCR program was 95 °C for 30 s and 45 cycles at 95 °C for 15 s, 62 °C for 30 s, and 72 °C for 20 s. The relative EGFP transcription levels were analyzed using the 2^–△△Ct^ method.^33^ The 16S rRNA was used as the endogenous reference gene, and the transcription level of EGFP in the plasmid containing a monocistronic promoter was defined as 1.

### Protein Preparation and SDS-PAGE Analysis

After 36 h of culturing in the 24-deep-well plate, the medium supernatants containing the heterologous protein were harvested by centrifugation at 12,000*g* for 10 min at 4 °C. The 10 μL protein samples were analyzed by 12% (w/v) sodium dodecyl sulfate-polyacrylamide gel electrophoresis (SDS-PAGE). After electrophoresis, the gels were stained with Coomassie blue (1 g Coomassie blue R-250, 10% (v/v) acetic acid, and 50% (v/v) methanol per liter) and destained using a decolorizing solution (5% (v/v) ethanol and 10% (v/v) acetic acid per liter). The relative intensity of target protein bands was quantified using ImageJ software.

### β-Galactosidase Assay

*C. glutamicum* cells were cultivated overnight in LBB medium; 500 μL of the cells were harvested by centrifugation and resuspended in 500 μL of ice-cooled Z buffer (60 mM Na_2_HPO_4_, 40 mM NaH_2_PO_4_·H_2_O, 10 mM KCl, 1 mM MgSO_4_, 50 mM β-mercaptoethanol, pH 7.0). The cell density was evaluated by determining the OD_600_. 100 μL samples were then taken out and mixed with 100 μL of chloroform and 50 μL of 0.1% SDS on a vortex for 10 s. After 1 h of incubation at 30 °C, 200 μL of ONPG (4 mg/mL) was added, and the samples were placed at 30 °C for further incubation. The reaction was stopped with 500 μL 1 M sodium carbonate. The A_420_ and A_550_ values were measured, and the β-galactosidase was calculated using the method described previously.^52−54^

### Construction of Fore-Cistron Library in *C. glutamicum*

Randomization of the fore-cistron sequence was achieved by using a primer, library-F, containing 59 bp of degenerate oligonucleotide N. The BCD expression cassette P_H36_-HP-6 was used as a template for PCR amplification, and a linearized plasmid fragment was obtained using the primers library-F/library-R. To avoid interference from the template plasmid, only 1 ng of plasmid was added to a 100 μL PCR reaction. After digestion with *Dpn*I, this purified PCR fragment was directly transformed into *E. coli* JM109. All transformants on solid plates were eluted and cultivated in a liquid LB medium for plasmid extraction. Finally, the purified plasmid library was transformed into *C. glutamicum* through electroporation.

### NGS Library Preparation, Sequencing, and Data Processing

The plasmid library from *C. glutamicum* cells was extracted using the kit from CWBIO (CW0500). The extracted plasmid was used as a template, and fore-cistron fragments (ca. 220 bp) were obtained by 20 cycles of amplification using a high-fidelity enzyme (Vazyme, P520-01) and the primers NGS-F/NGS-R. A sequencing library was then generated using the NEBNext Ultra DNA Library Prep Kit. The final library was sequenced on Illumina NovaSeq at GENEWIZ. For analysis, Cutadapt (version 1.9.1) was used to trim and filter the raw reads to remove sequences with low quality.^55^ Then, the clean data were merged by Pandaseq (version 2.7), and the polymorphism statistics were performed on merged reads of the library.^56^

### High-throughput Screening of the Fore-Cistron Library

The fore-cistron library of *C. glutamicum* was inoculated into LBB medium containing 10 mg/L chloramphenicol and cultivated at 30 °C for 12 h with shaking at 220 rpm. Then, the 1% (v/v) fraction of cells was transferred to the fresh LBB medium and grown for an additional 12 h. Cells were collected by centrifugation and washed three times with sterile phosphate-buffered saline (PBS). Finally, the cells were resuspended in PBS, and the OD_600_ was diluted to about 0.15. The library was analyzed and sorted using a fluorescence-activated cell sorter (FACS) Aria III (BD Biosciences, Franklin Lakes, NJ) based on fluorescence intensity detection through a 488/530 band-pass filter for the EGFP reporter. For cell sorting, FSC-A/FSC-H and SSC-A/SSC-H operation was executed to remove cell adhesion. The screened cells were then coated on LBHIS plates containing 10 mg/L chloramphenicol and cultivated for 48 h.

## Supporting Information Available

The relative transcriptional level of EGFP in leaderless BCDs (Figure S1), the effect of start codon mutation in leaderless BCDs on the fluorescence intensity and transcription level of EGFP (Figure S2), the effect of SD mutation in leaderless BCDs on the fluorescence intensity and transcription level of EGFP (Figure S3), verification of the reliability of the design principle (Figure S4). Bacteria strains and plasmids used in this study (Table S1), primers used in this study (Table S2), identification of transcription initiation sites (TSPs) of leaderless BCDs (Table S3), the fore-cistron sequence of 42 clones randomly selected from the H-group (Table S4) (PDF)
List of fore-cistron sequences in the original library (Table S5) (XLS)

## Notes

This article does not contain any study with human participants or animals by any of the authors.
