# Supplementary material for: Leaderless Bicistronic Design for Precise and Reliable Control of Gene Expression in Corynebacterium Glutamicum
Source: ACS Synth Biol. 2023 Jun 23;12(7):2157–67. doi: 10.1021/acssynbio.3c00246 (PMC10367133; doi:10.1021/acssynbio.3c00246)
Supplement: Supplementary file 1 — sb3c00246_si_001.pdf [file sb3c00246_si_001.pdf]

# Supporting Information

**Title：Leaderless Bicistronic Design for Precise and Reliable Control of Gene Expression in *Corynebacterium Glutamicum***

Xiuxia Liu[1,2,3 #], Manman Sun[1,4* #], Alex Xiong Gao[5 #], Rodrigo Ledesma-Amaro[4], Qiuwu Fang[6], Yankun Yang[1,2,3], Zhonghu Bai[1,2,3*]

[#] Co-first author

* Correspondence: manman.sun@imperial.ac.uk, baizhonghu@jiangnan.edu.cn

[1] National Engineering Research Center of Cereal Fermentation and Food Biomanufacturing, Jiangnan University, Wuxi 214112, China

[2] Key Laboratory of Industrial Biotechnology, Ministry of Education, School of Biotechnology, Jiangnan University, Wuxi 214122, China

[3] Jiangsu Provincial Research Center for Bioactive Product Processing Technology, Jiangnan University, Wuxi, 214122, China

[4] Department of Bioengineering and Imperial College Centre for Synthetic Biology, Imperial College London, London SW7 2AZ, UK

[5] Division of Life Science, The Hong Kong University of Science and Technology, Hong Kong, China

[6] Living Systems Institute, University of Exeter

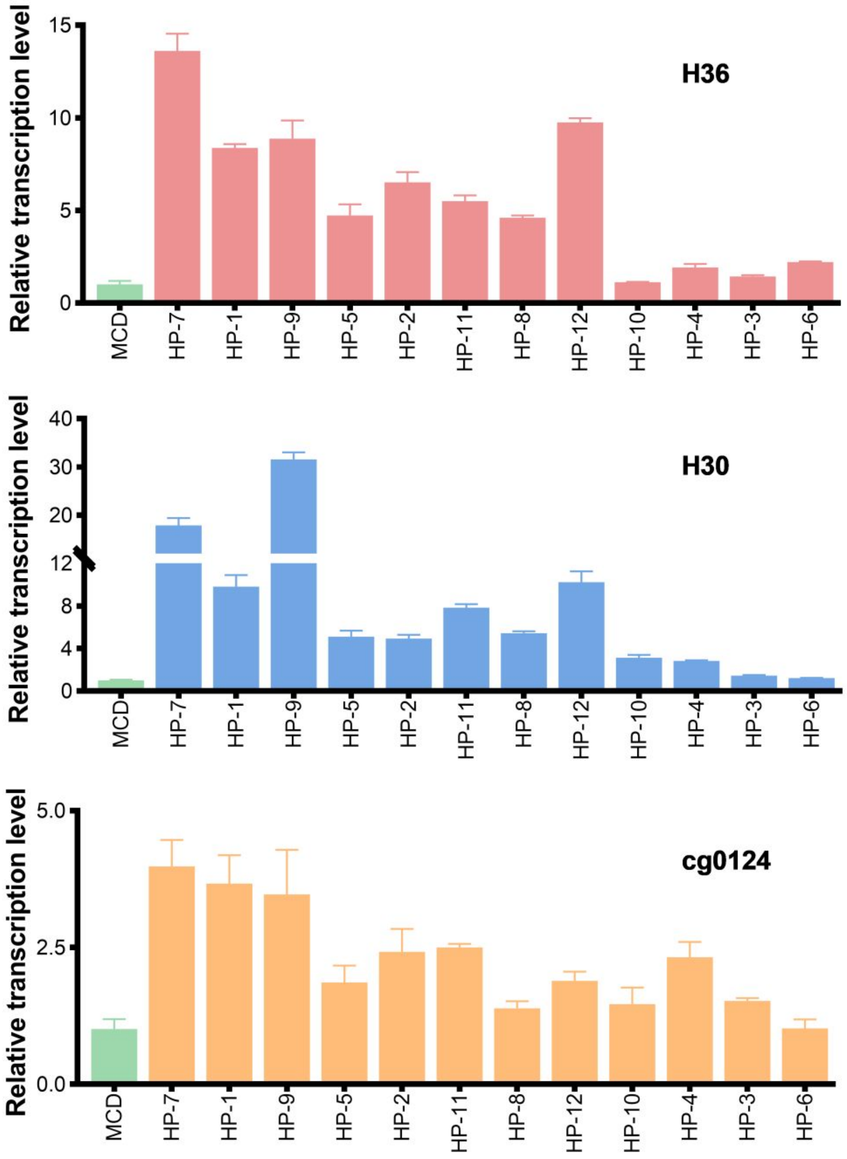


**Figure. S1 The relative transcriptional level of EGFP in leaderless BCDs.**

The EGFP transcription level under the control of the monocistronnic promoter (MCD) was defined as 1. HP-1 to HP-12 represented leaderless BCD containing different fore-cistron sequences.

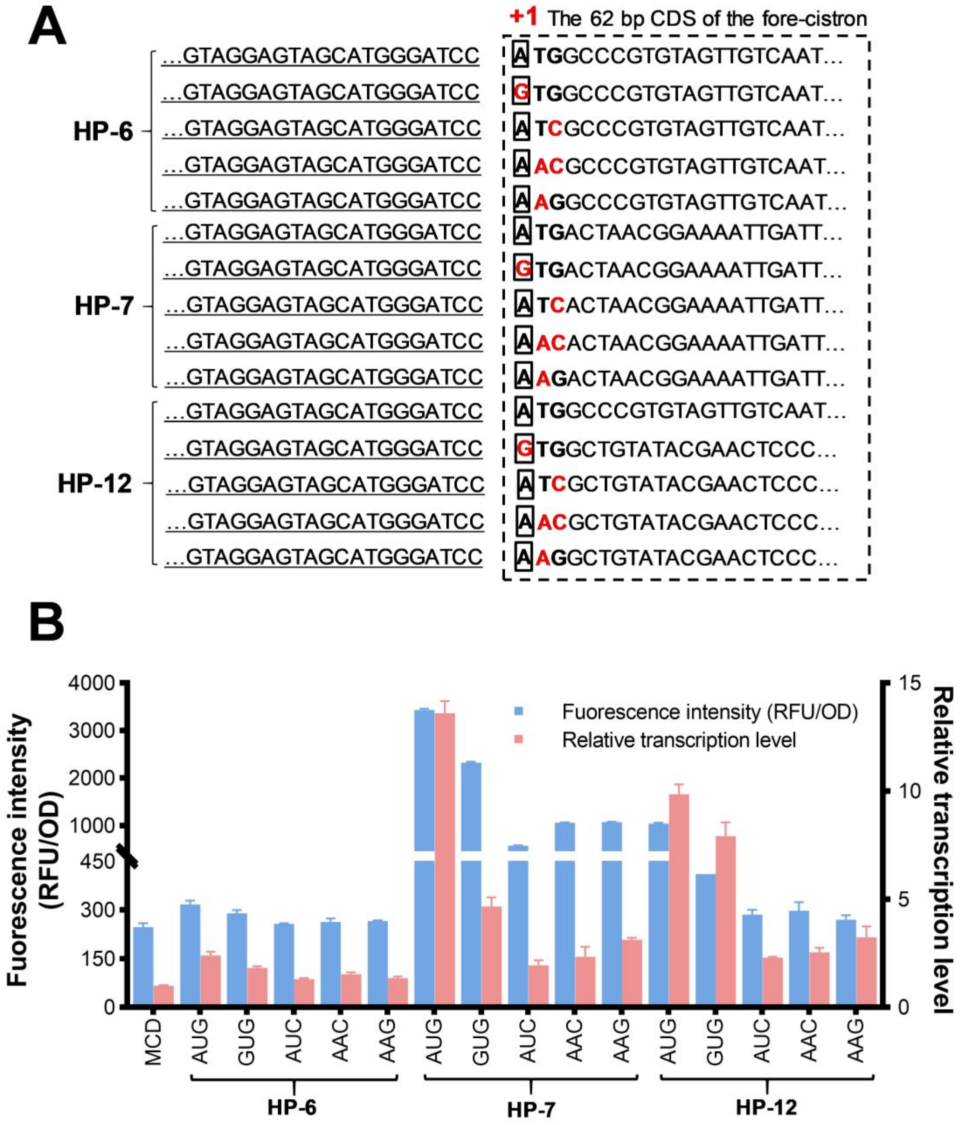


**Figure. S2 The effect of start codon mutation in leaderless BCDs on the fluorescence intensity and transcription level of EGFP.**

(A) Identification of transcription initiation sites (TSPs) of mutant. 5′RACE determined TSPs (+1) were given in bold letters with black boxes. The first codon triplet was shown in boldface, and the mutated base was shown in red characters. The underlined sequence represented the sequence of $P_{H36}$ and the dashed boxes indicated the fore-cistron sequence. (B) Effects of fore-cistron start codon mutation on the fluorescence intensity and transcription level of EGFP in leaderless BCDs.

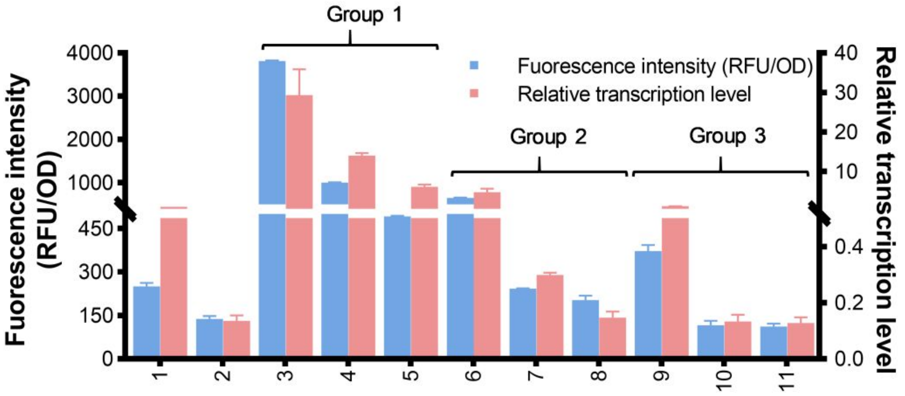


| | MCD | | BCD | | | | | | | | |
|---|---|---|---|---|---|---|---|---|---|---|---|
| Fore-cistron(AUC) | - | - | - | - | - | + | + | + | + | + | + |
| EGFP(AUC) | - | + | - | - | - | - | - | - | + | + | + |
| SD(ACCAGG) | - | - | - | + | - | - | + | - | - | + | - |
| SD(ACCACC) | - | - | - | - | + | - | - | + | - | - | + |

**Figure. S3 The effect of SD mutation in leaderless BCDs on the fluorescence intensity and transcription level of EGFP.**

The abscissa 1-2 represents the monocistronic expression cassette of $P_{H36}$, 3-11 represents the bicistronic expression cassette of $P_{H36}$. Group 1: BCD containing the HP-7 fore-cistron sequence; Group 2: BCD containing mutated HP-7 fore-cistron sequence; Group 3: BCD containing mutated HP-7 fore-cistron sequence and mutated EGFP gene. The red character represents the mutated base. The SD(ACCAGG) represents the SD motif of AAAGGAGGACAAC was mutated to AAACCAGGACAAC. The SD(ACCACC) represents the SD motif of AAAGGAGGACAAC was mutated to AAACCACCACAAC.

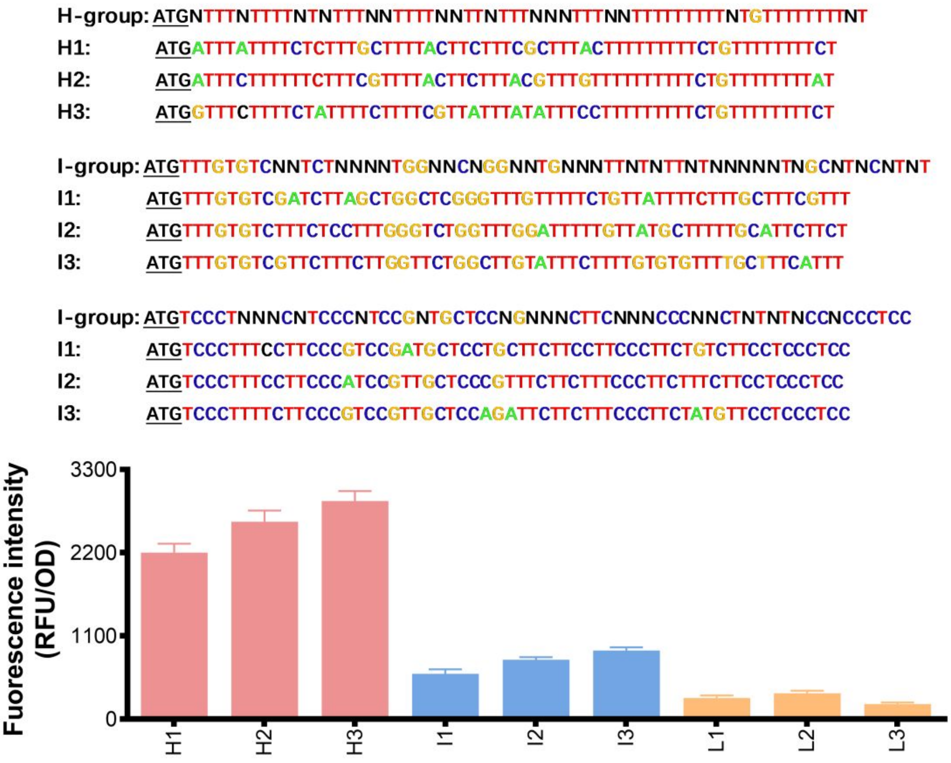


**Figure. S4 Verification of the reliability of the design principle.**

Three sequences randomly designed for each group and their corresponding fluorescence intensity

**Table S1** Bacteria strains and plasmids used in this study.

| Strains or plasmids | Description | Sources |
|---|---|---|
| **Strains** | | |
| *E. coli* JM109 | \ | |
| *C. glutamicum* CGMCC1.15647 | Wild type | (Sun et al. 2022) |
| *C. glutamicum* ATCC13032 | Wild type | (Sun et al. 2020) |
| **Plasmids** | | |
| pEC-XK99E | Providing a template to amplify the promoter $P_{aph}$ | (Zhang et al. 2017) |
| pXMJ19-EGFP | containing a monocistronic tac promoter and the EGFP gene | (Sun et al. 2020) |
| pXMJ19-VHH | containing a monocistronic tac promoter, CspB signal peptide, and the VHH gene | (Sun et al. 2022) |
| $P_{btac}$-HP-1 | containing a bicistronic tac promoter and the EGFP gene | (Sun et al. 2020) |
| $P_{btac}$-HP-2 | containing a bicistronic tac promoter and the EGFP gene | (Sun et al. 2020) |
| $P_{btac}$-HP-3 | containing a bicistronic tac promoter and the EGFP gene | (Sun et al. 2020) |
| $P_{btac}$-HP-4 | containing a bicistronic tac promoter and the EGFP gene | (Sun et al. 2020) |
| $P_{btac}$-HP-5 | containing a bicistronic tac promoter and the EGFP gene | (Sun et al. 2020) |
| $P_{btac}$-HP-6 | containing a bicistronic tac promoter and the EGFP gene | (Sun et al. 2020) |
| $P_{btac}$-HP-7 | containing a bicistronic tac promoter and the EGFP gene | (Sun et al. 2020) |
| $P_{btac}$-HP-8 | containing a bicistronic tac promoter and the EGFP gene | (Sun et al. 2020) |
| $P_{btac}$-HP-9 | containing a bicistronic tac promoter and the EGFP gene | (Sun et al. 2020) |
| $P_{btac}$-HP-10 | containing a bicistronic tac promoter and the EGFP gene | (Sun et al. 2020) |
| $P_{btac}$-HP-11 | containing a bicistronic tac promoter and the EGFP gene | (Sun et al. 2020) |
| $P_{btac}$-HP-12 | containing a bicistronic tac promoter and the EGFP gene | (Sun et al. 2020) |
| $P_{H36}$-MCD | containing a monocistronic H36 promoter and the EGFP gene | This study |
| $P_{H36}$-HP-1 | containing a bicistronic H36 promoter and the EGFP gene | This study |
| $P_{H36}$-HP-2 | containing a bicistronic H36 promoter and the EGFP gene | This study |
| $P_{H36}$-HP-3 | containing a bicistronic H36 promoter and the EGFP gene | This study |
| $P_{H36}$-HP-4 | containing a bicistronic H36 promoter and the EGFP gene | This study |
| $P_{H36}$-HP-5 | containing a bicistronic H36 promoter and the EGFP gene | This study |
| $P_{H36}$-HP-6 | containing a bicistronic H36 promoter and the EGFP gene | This study |
| $P_{H36}$-HP-7 | containing a bicistronic H36 promoter and the EGFP gene | This study |
| $P_{H36}$-HP-8 | containing a bicistronic H36 promoter and the EGFP gene | This study |
| $P_{H36}$-HP-9 | containing a bicistronic H36 promoter and the EGFP gene | This study |
| $P_{H36}$-HP-10 | containing a bicistronic H36 promoter and the EGFP gene | This study |
| $P_{H36}$-HP-11 | containing a bicistronic H36 promoter and the EGFP gene | This study |
| $P_{H36}$-HP-12 | containing a bicistronic H36 promoter and the EGFP gene | This study |
| $P_{H30}$-MCD | containing a monocistronic H30 promoter and the EGFP gene | This study |
| $P_{H30}$-HP-1 | containing a bicistronic H30 promoter and the EGFP gene | This study |

**Table S1.** continued.

| Strains or plasmids | Description | Sources |
|---|---|---|
| $P_{H30}$-HP-2 | containing a bicistronic H30 promoter and the EGFP gene | This study |
| $P_{H30}$-HP-3 | containing a bicistronic H30 promoter and the EGFP gene | This study |
| $P_{H30}$-HP-4 | containing a bicistronic H30 promoter and the EGFP gene | This study |
| $P_{H30}$-HP-5 | containing a bicistronic H30 promoter and the EGFP gene | This study |
| $P_{H30}$-HP-6 | containing a bicistronic H30 promoter and the EGFP gene | This study |
| $P_{H30}$-HP-7 | containing a bicistronic H30 promoter and the EGFP gene | This study |
| $P_{H30}$-HP-8 | containing a bicistronic H30 promoter and the EGFP gene | This study |
| $P_{H30}$-HP-9 | containing a bicistronic H30 promoter and the EGFP gene | This study |
| $P_{H30}$-HP-10 | containing a bicistronic H30 promoter and the EGFP gene | This study |
| $P_{H30}$-HP-11 | containing a bicistronic H30 promoter and the EGFP gene | This study |
| $P_{H30}$-HP-12 | containing a bicistronic H30 promoter and the EGFP gene | This study |
| $P_{cg0124}$-MCD | containing a monocistronic cg0124 promoter and the EGFP gene | This study |
| $P_{cg0124}$-HP-1 | containing a bicistronic cg0124 promoter and the EGFP gene | This study |
| $P_{cg0124}$-HP-2 | containing a bicistronic cg0124 promoter and the EGFP gene | This study |
| $P_{cg0124}$-HP-3 | containing a bicistronic cg0124 promoter and the EGFP gene | This study |
| $P_{cg0124}$-HP-4 | containing a bicistronic cg0124 promoter and the EGFP gene | This study |
| $P_{cg0124}$-HP-5 | containing a bicistronic cg0124 promoter and the EGFP gene | This study |
| $P_{cg0124}$-HP-6 | containing a bicistronic cg0124 promoter and the EGFP gene | This study |
| $P_{cg0124}$-HP-7 | containing a bicistronic cg0124 promoter and the EGFP gene | This study |
| $P_{cg0124}$-HP-8 | containing a bicistronic cg0124 promoter and the EGFP gene | This study |
| $P_{cg0124}$-HP-9 | containing a bicistronic cg0124 promoter and the EGFP gene | This study |
| $P_{cg0124}$-HP-10 | containing a bicistronic cg0124 promoter and the EGFP gene | This study |
| $P_{cg0124}$-HP-11 | containing a bicistronic cg0124 promoter and the EGFP gene | This study |
| $P_{cg0124}$-HP-12 | containing a bicistronic cg0124 promoter and the EGFP gene | This study |
| $P_{aph}$-MCD | containing a monocistronic aph promoter and the EGFP gene | This study |
| $P_{aph}$-HP-1 | containing a bicistronic aph promoter and the EGFP gene | This study |
| $P_{aph}$-HP-2 | containing a bicistronic aph promoter and the EGFP gene | This study |
| $P_{aph}$-HP-3 | containing a bicistronic aph promoter and the EGFP gene | This study |
| $P_{aph}$-HP-4 | containing a bicistronic aph promoter and the EGFP gene | This study |
| $P_{aph}$-HP-5 | containing a bicistronic aph promoter and the EGFP gene | This study |
| $P_{aph}$-HP-6 | containing a bicistronic aph promoter and the EGFP gene | This study |
| $P_{aph}$-HP-7 | containing a bicistronic aph promoter and the EGFP gene | This study |
| $P_{aph}$-HP-8 | containing a bicistronic aph promoter and the EGFP gene | This study |
| $P_{aph}$-HP-9 | containing a bicistronic aph promoter and the EGFP gene | This study |
| $P_{aph}$-HP-10 | containing a bicistronic aph promoter and the EGFP gene | This study |
| $P_{aph}$-HP-11 | containing a bicistronic aph promoter and the EGFP gene | This study |
| $P_{aph}$-HP-12 | containing a bicistronic aph promoter and the EGFP gene | This study |

**Table S1.** continued.

| Strains or plasmids | Description | Sources |
|---|---|---|
| $P_{tuf}$-MCD | containing a monocistronic tuf promoter and the EGFP gene | This study |
| $P_{tuf}$-HP-1 | containing a bicistronic tuf promoter and the EGFP gene | This study |
| $P_{tuf}$-HP-2 | containing a bicistronic tuf promoter and the EGFP gene | This study |
| $P_{tuf}$-HP-3 | containing a bicistronic tuf promoter and the EGFP gene | This study |
| $P_{tuf}$-HP-4 | containing a bicistronic tuf promoter and the EGFP gene | This study |
| $P_{tuf}$-HP-5 | containing a bicistronic tuf promoter and the EGFP gene | This study |
| $P_{tuf}$-HP-6 | containing a bicistronic tuf promoter and the EGFP gene | This study |
| $P_{tuf}$-HP-7 | containing a bicistronic tuf promoter and the EGFP gene | This study |
| $P_{tuf}$-HP-8 | containing a bicistronic tuf promoter and the EGFP gene | This study |
| $P_{tuf}$-HP-9 | containing a bicistronic tuf promoter and the EGFP gene | This study |
| $P_{tuf}$-HP-10 | containing a bicistronic tuf promoter and the EGFP gene | This study |
| $P_{tuf}$-HP-11 | containing a bicistronic tuf promoter and the EGFP gene | This study |
| $P_{tuf}$-HP-12 | containing a bicistronic tuf promoter and the EGFP gene | This study |
| $P_{H36}$-MCD-VHH | containing a monocistronic H36 promoter, CspB signal peptide, and the VHH gene | This study |
| $P_{H36}$-HP-6-VHH | containing a bicistronic H36 promoter, CspB signal peptide, and the VHH gene | This study |
| $P_{H36}$-HP-7-VHH | containing a bicistronic H36 promoter, CspB signal peptide, and the VHH gene | This study |
| $P_{H36}$-HP-12-VHH | containing a bicistronic H36 promoter, CspB signal peptide, and the VHH gene | This study |
| $P_{H30}$-HP-7-VHH | containing a bicistronic H30 promoter, CspB signal peptide, and the VHH gene | This study |
| $P_{cg0124}$-HP-7-VHH | containing a bicistronic cg0124 promoter, CspB signal peptide, and the VHH gene | This study |
| $P_{H36}$-MCD-LacZ | containing a monocistronic H36 promoter and the LacZ gene | This study |
| $P_{H36}$-HP-6-LacZ | containing a bicistronic H36 promoter and the LacZ gene | This study |
| $P_{H36}$-HP-7-LacZ | containing a bicistronic H36 promoter and the LacZ gene | This study |
| $P_{H36}$-HP-12-LacZ | containing a bicistronic H36 promoter and the LacZ gene | This study |
| $P_{H30}$-HP-7-LacZ | containing a bicistronic H30 promoter and the LacZ gene | This study |
| $P_{cg0124}$-HP-7-LacZ | containing a bicistronic cg0124 promoter and the LacZ gene | This study |

**Table S2 Primers used in this study**

| Primer | Sequence (5′-3′)[a] | Intension |
|---|---|---|
| $P_{H36}$-F | GTATCCCACTACCGAGATATCCAAAAGCTGGGTACCTCTATCTGGTGCCCTAAACGGGG GAATATTAACGGGCCCA | Amplifying $P_{H36}$ |
| $P_{H36}$-EGFP-BR | GGATCCCATGCTACTCCTACCAACCAAGGTGCGACCACCCTGGGCCCGTTAATATTCCC CC | Amplifying $P_{H36}$ for BCD containing a EGFP gene |
| $P_{H36}$-EGFP-MR | TCCTCGCCCTTGCTCACCATGGATCCCATGCTACTCCTACCAACCAAGGTGCGACCACC CTGGGCCCGTTAATATTCCCCC | Amplifying $P_{H36}$ for MCD containing a EGFP gene |
| $P_{H30}$-F | TCGTCGTATCCCACTACCGAGATATCCAAAAGCTGGGTACCAAAGTAACTTTTCGGTTA AGGTAGCGCATTCGTGGTGTTGC | Amplifying $P_{H30}$ |
| $P_{H30}$-EGFP-BR | GGATCCCAATATACTCCTGCCCAACCAACCGGGCCACGGGCAACACCACGAATGCGCT ACC | Amplifying $P_{H30}$ for BCD containing a EGFP gene |
| $P_{H30}$-EGFP-MR | TCCTCGCCCTTGCTCACCATGGATCCCAATATACTCCTGCCCAACCAACCGGGCCACGG GCAACACCACGAATGCGCTACCT | Amplifying $P_{H30}$ for MCD containing a EGFP gene |
| $P_{Cg0124}$-F | GTATCCCACTACCGAGATATCAACTTGGAAATTATCACGTGAGGATTCTTGCATACGCC TATAAAAGCACAGTTTTGAATCCACA | Amplifying $P_{Cg0124}$ |
| $P_{Cg0124}$-BR | GCGCACCACACTATCTTTCTGCACGCCCTGATGCCCTGTGGATTCAAAACTGTGCT | Amplifying $P_{Cg0124}$ for BCD containing a EGFP gene |
| $P_{Cg0124}$-MR | TCCTCGCCCTTGCTCACCATGCGCACCACACTATCTTTCTGCACGCCCTGATGCCCTGTG GATTCAAAACTGTGCT | Amplifying $P_{Cg0124}$ for MCD containing a EGFP gene |
| $P_{aph}$-F | CGTCGTATCCCACTACCGAGATATCAGCTTCACGCTGCCGCAAGCA | Amplifying $P_{aph}$ |
| $P_{aph}$-BR | GCGAAACGATCCTCATCCTGT | Amplifying $P_{aph}$ for BCD |
| $P_{aph}$-MR | TCCTCGCCCTTGCTCACCATGCGAAACGATCCTCATCCTGT | Amplifying $P_{aph}$ for MCD |
| $P_{tuf}$-F | GTCGTATCCCACTACCGAGATATCTTTCAACAGCATTGATTTCGA | Amplifying $P_{tuf}$ |
| $P_{tuf}$-BR | TGTATGTCCTCCTGGACTTCGT | Amplifying $P_{tuf}$ for BCD |
| $P_{tuf}$-MR | TCCTCGCCCTTGCTCACCATTGTATGTCCTCCTGGACTTCGT | Amplifying $P_{tuf}$ for MCD |
| $H_{36}$-HP-1-F | TAGGAGTAGCATGGGATCCATGGCAAACGTCAACATCAAGCCGCTTGAGGACAAGATC CTCGTTCAGAT | Amplifying HP-1 for $P_{H36}$ |
| $H_{36}$-HP-2-F | TAGGAGTAGCATGGGATCCATGACTGAACGTACTCTCATCCTTATCAAGCCAGACGGTG TTACCAACGGA | Amplifying HP-2 for $P_{H36}$ |
| $H_{36}$-HP-3-F | TAGGAGTAGCATGGGATCCATGCCTATCGCAACTCCCGAGGTCTATAACGAGATGCTCG ATCGTGCTAAG | Amplifying HP-3 for $P_{H36}$ |
| $H_{36}$-HP-4-F | TAGGAGTAGCATGGGATCCATGGGGTCCATGGCTAAAACACATTTTCAAGGCAACGAA ACTGCTACCT | Amplifying HP-4 for $P_{H36}$ |
| $H_{36}$-HP-5-F | TAGGAGTAGCATGGGATCCATGAGCGAGAATTACAGCAAGATTGTCGTTGGCACTGAT GGATCTAAGT | Amplifying HP-5 for $P_{H36}$ |
| $H_{36}$-HP-6-F | TAGGAGTAGCATGGGATCCATGGCCCGTGTAGTTGTCAATGTCATGCCTAAGGCTGAGA TTCTGGAT | Amplifying HP-6 for $P_{H36}$ |
| $H_{36}$-HP-7-F | TAGGAGTAGCATGGGATCCATGACTAACGGAAAATTGATTCTTCTTCGTCACGGTCAGA GCGAATGGAA | Amplifying HP-7 for $P_{H36}$ |
| $H_{36}$-HP-8-F | TAGGAGTAGCATGGGATCCATGGCTGAAATCATGCACGTATTCGCTCGCGAAATTCTCG ACTCCCGCGGT | Amplifying HP-8 for $P_{H36}$ |
| $H_{36}$-HP-9-F | TAGGAGTAGCATGGGATCCATGAGCGATATTCGTATGGCAGCCCAGGGTGGGCCTGGT TTCGGAAATGA | Amplifying HP-9 for $P_{H36}$ |

**Table S2.** continued.

| Primer | Sequence (5′-3′)[a] | Intension |
|---|---|---|
| $H_{36}$-HP-10-F | TAGGAGTAGCATGGGATCCATGCGACTCGTACTCCTCGGACCTCCCGGTGCTGGTAAGG GCACCCAGGCT | Amplifying HP-10 for $P_{H36}$ |
| $H_{36}$-HP-11-F | TAGGAGTAGCATGGGATCCATGATTGGAGCACCACCCGACATGGGCAATGTGTACAAC AACATCACCGA | Amplifying HP-11 for $P_{H36}$ |
| $H_{36}$-HP-12-F | TAGGAGTAGCATGGGATCCATGGCTGTATACGAACTCCCAGAACTCGACTACGCATAC GACGCTCTCGA | Amplifying HP-12 for $P_{H36}$ |
| $H_{30}$-HP-1-F | GCAGGAGTATATTGGGATCCATGGCAAACGTCAACATCAAGCCGCTTGAGGACAAGAT CCTCGTTCAGAT | Amplifying HP-1 for $P_{H30}$ |
| $H_{30}$-HP-2-F | GCAGGAGTATATTGGGATCCATGACTGAACGTACTCTCATCCTTATCAAGCCAGACGGT GTTACCAACGGA | Amplifying HP-2 for $P_{H30}$ |
| $H_{30}$-HP-3-F | GCAGGAGTATATTGGGATCCATGCCTATCGCAACTCCCGAGGTCTATAACGAGATGCTC GATCGTGCTAAG | Amplifying HP-3 for $P_{H30}$ |
| $H_{30}$-HP-4-F | GCAGGAGTATATTGGGATCCATGGGGTCCATGGCTAAAACACATTTTCAAGGCAACGA AACTGCTACCT | Amplifying HP-4 for $P_{H30}$ |
| $H_{30}$-HP-5-F | GCAGGAGTATATTGGGATCCATGAGCGAGAATTACAGCAAGATTGTCGTTGGCACTGA TGGATCTAAGT | Amplifying HP-5 for $P_{H30}$ |
| $H_{30}$-HP-6-F | GCAGGAGTATATTGGGATCCATGGCCCGTGTAGTTGTCAATGTCATGCCTAAGGCTGAG ATTCTGGAT | Amplifying HP-6 for $P_{H30}$ |
| $H_{30}$-HP-7-F | GCAGGAGTATATTGGGATCCATGACTAACGGAAAATTGATTCTTCTTCGTCACGGTCAG AGCGAATGGAA | Amplifying HP-7 for $P_{H30}$ |
| $H_{30}$-HP-8-F | GCAGGAGTATATTGGGATCCATGGCTGAAATCATGCACGTATTCGCTCGCGAAATTCTC GACTCCCGCGGT | Amplifying HP-8 for $P_{H30}$ |
| $H_{30}$-HP-9-F | GCAGGAGTATATTGGGATCCATGAGCGATATTCGTATGGCAGCCCAGGGTGGGCCTGG TTTCGGAAATGA | Amplifying HP-9 for $P_{H30}$ |
| $H_{30}$-HP-10-F | GCAGGAGTATATTGGGATCCATGCGACTCGTACTCCTCGGACCTCCCGGTGCTGGTAAG GGCACCCAGGCT | Amplifying HP-10 for $P_{H30}$ |
| $H_{30}$-HP-11-F | GCAGGAGTATATTGGGATCCATGATTGGAGCACCACCCGACATGGGCAATGTGTACAA CAACATCACCGA | Amplifying HP-11 for $P_{H30}$ |
| $H_{30}$-HP-12-F | GCAGGAGTATATTGGGATCCATGGCTGTATACGAACTCCCAGAACTCGACTACGCATAC GACGCTCTCGA | Amplifying HP-12 for $P_{H30}$ |
| cg0124-HP-1-F | AGAAAGATAGTGTGGTGCGCATGGCAAACGTCAACATCAAGCCGCTTGAGGACAAGAT CCTCGTTCAGAT | Amplifying HP-1 for $P_{cg0124}$ |
| cg0124-HP-2-F | AGAAAGATAGTGTGGTGCGCATGACTGAACGTACTCTCATCCTTATCAAGCCAGACGGT GTTACCAACGGA | Amplifying HP-2 for $P_{cg0124}$ |
| cg0124-HP-3-F | AGAAAGATAGTGTGGTGCGCATGCCTATCGCAACTCCCGAGGTCTATAACGAGATGCT CGATCGTGCTAAG | Amplifying HP-3 for $P_{cg0124}$ |
| cg0124-HP-4-F | AGAAAGATAGTGTGGTGCGCATGGGGTCCATGGCTAAAACACATTTTCAAGGCAACGA AACTGCTACCT | Amplifying HP-4 for $P_{cg0124}$ |
| cg0124-HP-5-F | AGAAAGATAGTGTGGTGCGCATGAGCGAGAATTACAGCAAGATTGTCGTTGGCACTGA TGGATCTAAGT | Amplifying HP-5 for $P_{cg0124}$ |
| cg0124-HP-6-F | AGAAAGATAGTGTGGTGCGCATGGCCCGTGTAGTTGTCAATGTCATGCCTAAGGCTGA GATTCTGGAT | Amplifying HP-6 for $P_{cg0124}$ |

**Table S2.** continued.

| Primer | Sequence (5′-3′)[a] | Intension |
|---|---|---|
| cg0124-HP-7-F | AGAAAGATAGTGTGGTGCGCATGACTAACGGAAAATTGATTCTTCTTCGTCACGGTCAGAGCGAATGGAA | Amplifying HP-7 for $P_{cg0124}$ |
| cg0124-HP-8-F | AGAAAGATAGTGTGGTGCGCATGGCTGAAATCATGCACGTATTCGCTCGCGAAATTCTCGACTCCCGCGGT | Amplifying HP-8 for $P_{cg0124}$ |
| cg0124-HP-9-F | AGAAAGATAGTGTGGTGCGCATGAGCGATATTCGTATGGCAGCCCAGGGTGGGCCTGGTTTCGGAAATGA | Amplifying HP-9 for $P_{cg0124}$ |
| cg0124-HP-10-F | AGAAAGATAGTGTGGTGCGCATGCGACTCGTACTCCTCGGACCTCCCGGTGCTGGTAAGGGCACCCAGGCT | Amplifying HP-10 for $P_{cg0124}$ |
| cg0124-HP-11-F | AGAAAGATAGTGTGGTGCGCATGATTGGAGCACCACCCGACATGGGCAATGTGTACAACAACATCACCGA | Amplifying HP-11 for $P_{cg0124}$ |
| cg0124-HP-12-F | AGAAAGATAGTGTGGTGCGCATGGCTGTATACGAACTCCCAGAACTCGACTACGCATACGACGCTCTCGA | Amplifying HP-12 for $P_{cg0124}$ |
| aph-HP-1-F | CAGGATGAGGATCGTTTCGCATGGCAAACGTCAACATCAAGCCGCTTGAGGACAAGATCCTCGTTCAGAT | Amplifying HP-1 for $P_{aph}$ |
| aph-HP-2-F | CAGGATGAGGATCGTTTCGCATGACTGAACGTACTCTCATCCTTATCAAGCCAGACGGTGTTACCAACGGA | Amplifying HP-2 for $P_{aph}$ |
| aph-HP-3-F | CAGGATGAGGATCGTTTCGCATGCCTATCGCAACTCCCGAGGTCTATAACGAGATGCTCGATCGTGCTAAG | Amplifying HP-3 for $P_{aph}$ |
| aph-HP-4-F | CAGGATGAGGATCGTTTCGCATGGGGTCCATGGCTAAAACACATTTTCAAGGCAACGAAACTGCTACCT | Amplifying HP-4 for $P_{aph}$ |
| aph-HP-5-F | CAGGATGAGGATCGTTTCGCATGAGCGAGAATTACAGCAAGATTGTCGTTGGCACTGATGGATCTAAGT | Amplifying HP-5 for $P_{aph}$ |
| aph-HP-6-F | CAGGATGAGGATCGTTTCGCATGGCCCGTGTAGTTGTCAATGTCATGCCTAAGGCTGAGATTCTGGAT | Amplifying HP-6 for $P_{aph}$ |
| aph-HP-7-F | CAGGATGAGGATCGTTTCGCATGACTAACGGAAAATTGATTCTTCTTCGTCACGGTCAGAGCGAATGGAA | Amplifying HP-7 for $P_{aph}$ |
| aph-HP-8-F | CAGGATGAGGATCGTTTCGCATGGCTGAAATCATGCACGTATTCGCTCGCGAAATTCTCGACTCCCGCGGT | Amplifying HP-8 for $P_{aph}$ |
| aph-HP-9-F | CAGGATGAGGATCGTTTCGCATGAGCGATATTCGTATGGCAGCCCAGGGTGGGCCTGGTTTCGGAAATGA | Amplifying HP-9 for $P_{aph}$ |
| aph-HP-10-F | CAGGATGAGGATCGTTTCGCATGCGACTCGTACTCCTCGGACCTCCCGGTGCTGGTAAGGGCACCCAGGCT | Amplifying HP-10 for $P_{aph}$ |
| aph-HP-11-F | CAGGATGAGGATCGTTTCGCATGATTGGAGCACCACCCGACATGGGCAATGTGTACAACAACATCACCGA | Amplifying HP-11 for $P_{aph}$ |
| aph-HP-12-F | CAGGATGAGGATCGTTTCGCATGGCTGTATACGAACTCCCAGAACTCGACTACGCATACGACGCTCTCGA | Amplifying HP-12 for $P_{aph}$ |
| tuf-HP-1-F | GAAGTCCAGGAGGACATACAATGGCAAACGTCAACATCAAGCCGCTTGAGGACAAGATCCTCGTTCAGAT | Amplifying HP-1 for $P_{tuf}$ |
| tuf-HP-2-F | GAAGTCCAGGAGGACATACAATGACTGAACGTACTCTCATCCTTATCAAGCCAGACGGTGTTACCAACGGA | Amplifying HP-2 for $P_{tuf}$ |
| tuf-HP-3-F | GAAGTCCAGGAGGACATACAATGCCTATCGCAACTCCCGAGGTCTATAACGAGATGCTCGATCGTGCTAAG | Amplifying HP-3 for $P_{tuf}$ |

**Table S2.** continued.

| Primer | Sequence (5′-3′)[a] | Intension |
|---|---|---|
| tuf-HP-4-F | GAAGTCCAGGAGGACATACAATGGGGTCCATGGCTAAAACACATTTTCAAGGCAACGAAACTGCTACCT | Amplifying HP-4 for $P_{tuf}$ |
| tuf-HP-5-F | GAAGTCCAGGAGGACATACAATGAGCGAGAATTACAGCAAGATTGTCGTTGGCACTGATGGATCTAAGT | Amplifying HP-5 for $P_{tuf}$ |
| tuf-HP-6-F | GAAGTCCAGGAGGACATACAATGGCCCGTGTAGTTGTCAATGTCATGCCTAAGGCTGAGATTCTGGAT | Amplifying HP-6 for $P_{tuf}$ |
| tuf-HP-7-F | GAAGTCCAGGAGGACATACAATGACTAACGGAAAATTGATTCTTCTTCGTCACGGTCAGAGCGAATGGAA | Amplifying HP-7 for $P_{tuf}$ |
| tuf-HP-8-F | GAAGTCCAGGAGGACATACAATGGCTGAAATCATGCACGTATTCGCTCGCGAAATTCTCGACTCCCGCGGT | Amplifying HP-8 for $P_{tuf}$ |
| tuf-HP-9-F | GAAGTCCAGGAGGACATACAATGAGCGATATTCGTATGGCAGCCCAGGGTGGGCCTGGTTTCGGAAATGA | Amplifying HP-9 for $P_{tuf}$ |
| tuf-HP-10-F | GAAGTCCAGGAGGACATACAATGCGACTCGTACTCCTCGGACCTCCCGGTGCTGGTAAGGGCACCCAGGCT | Amplifying HP-10 for $P_{tuf}$ |
| tuf-HP-11-F | GAAGTCCAGGAGGACATACAATGATTGGAGCACCACCCGACATGGGCAATGTGTACAACAACATCACCGA | Amplifying HP-11 for $P_{tuf}$ |
| tuf-HP-12-F | GAAGTCCAGGAGGACATACAATGGCTGTATACGAACTCCCAGAACTCGACTACGCATACGACGCTCTCGA | Amplifying HP-12 for $P_{tuf}$ |
| HP-1-R | TCCTCGCCCTTGCTCACCATCATTAGTTGTCCTCCTTTTCTGCTTCGTTGATCTGAACGAGGATC | Amplifying HP-1 |
| HP-2-R | TCCT**CGCCCTTGCTCAC**CATCATTAGTTGTCCTCCTTTTCGCCGACGTGTCCGTTGGTAACACCGTC | Amplifying HP-2 |
| HP-3-R | TCCTCGCCCTTGCTCACCATCATTAGTTGTCCTCCTTTAATCCGCCTTCCTTAGCACGATCGAGCAT | Amplifying HP-3 |
| HP-4-R | TCCTCGCCCTTGCTCACCATCATTAGTTGTCCTCCTTTGGCAGTTCGCCGGAGGTAGCAGTTTCGTTGCCT | Amplifying HP-4 |
| HP-5-R | TCCTCGCCCTTGCTCACCATCATTAGTTGTCCTCCTTTGCTAGAAGGGACGACTTAGATCCATCAGTGCCA | Amplifying HP-5 |
| HP-6-R | TCCTCGCCCTTGCTCACCATCATTAGTTGTCCTCCTTTGCCTGCCCCTGGGGATCCAGAATCTCAGCCTT | Amplifying HP-6 |
| HP-7-R | TCCTCGCCCTTGCTCACCATCATTAGTTGTCCTCCTTTTGGTTGGATGCGTTCCATTCGCTCTGACCGTG | Amplifying HP-7 |
| HP-8-R | TCCTCGCCCTTGCTCACCATCATTAGTTGTCCTCCTTTACGGTTGGGTTACCGCGGGAGTCGAGAATTTC | Amplifying HP-8 |
| HP-9-R | TCCTCGCCCTTGCTCACCATCATTAGTTGTCCTCCTTTCGATCAAAGACGTCATTTCCGAAACCAGGCCCA | Amplifying HP-9 |
| HP-10-R | TCCTCGCCCTTGCTCACCATCATTAGTTGTCCTCCTTTGAGAGAATTGCAGCCTGGGTGCCCTTACCA | Amplifying HP-10 |
| HP-11-R | TCCTCGCCCTTGCTCACCATCATTAGTTGTCCTCCTTTTGGCCGATGGTTTCGGTGATGTTGTTGTAC | Amplifying HP-11 |
| HP-12-R | TCCTCGCCCTTGCTCACCATCATTAGTTGTCCTCCTTTGCGATGTGTGGCTCGAGAGCGTCGTATGCGT | Amplifying HP-12 |

**Table S2.** continued.

| Primer | Sequence (5′-3′)[a] | Intension |
|---|---|---|
| EGFP-F | ATGGTGAGCAAGGGCGAGGA | Amplifying EGFP |
| EGFP-R | TCCGCCAAAACAGCCAAGCTGAATTCTTACTTGTACAGCTCGTCCATGCCG | Amplifying EGFP |
| $P_{H36}$-VHH-BR | TGCGGATACGATTGTTAAACATCATTAGTTGTCCTCCTTT | Amplifying $H_{36}$ promoter and fore-cistron sequence for VHH |
| $P_{H36}$-VHH-MR | GTGCGGATACGATTGTTAAACATGGATCCCATGCTACTCCTACCA | Amplifying $H_{36}$ promoter for VHH |
| VHH-F | ATGTTTAACAATCGTATCCGCA | Amplifying CspB signal peptide and the VHH gene |
| VHH-R | CCAAAACAGCCAAGCTGAATTCTCAGTGGTGGTGGTGGTGGTGTGAAG | Amplifying the VHH gene |
| $P_{H36}$-LacZ-BR | TGAATCCGTAATCATGGTCATCATTAGTTGTCCTCCTTT | Amplifying $H_{36}$ promoter and fore-cistron sequence for LacZ |
| $P_{H36}$-LacZ-MR | GAATCCGTAATCATGGTCATGGATCCCATGCTACTCCTACCA | Amplifying $H_{36}$ promoter for LacZ |
| LacZ-F | ATGACCATGATTACGGATTCA | Amplifying the LacZ gene |
| LacZ-R | CCAAAACAGCCAAGCTGAATTCTTATTTTTGACACCAGACCAA | Amplifying the LacZ gene |
| MCD-mF1 | TTGGTTGGTAGGAGTAGCATGGGATCC**ATC**GTGAGCAAGGGCGAGGA | Mutating the "AUG" of EGFP into "AUC" for MCD |
| MCD- mF2 | TTGGTTGGTAGGAGTAGCATGGGATCC**ATCCTG**AGCAAGGGCGAGGA | Mutating the "AUGGUG" of EGFP into "AUCCUG" for MCD |
| HP-6-mF1 | TAGGAGTAGCATGGGATCC**ATC**GCCCGTGTAGTTGTCAATGTCATGCCTAAGGCTGAG ATTCTGGAT | Mutating the "AUG" of HP-6 into "AUC" |
| HP-6-mF2 | TAGGAGTAGCATGGGATCC**GTG**GCCCGTGTAGTTGTCAATGTCATGCCTAAGGCTGAG ATTCTGGAT | Mutating the "AUG" of HP-6 into "GUG" |
| HP-6-mF3 | TAGGAGTAGCATGGGATCC**AAC**GCCCGTGTAGTTGTCAATGTCATGCCTAAGGCTGAG ATTCTGGAT | Mutating the "AUG" of HP-6 into "AAC" |
| HP-6-mF4 | TAGGAGTAGCATGGGATCC**AAG**GCCCGTGTAGTTGTCAATGTCATGCCTAAGGCTGAG ATTCTGGAT | Mutating the "AUG" of HP-6 into "AAG" |
| HP-7-mF1 | TAGGAGTAGCATGGGATCC**ATC**AGCGAGAATTACAGCAAGATTGTCGTTGGCACTGAT GGATCT | Mutating the "AUG" of HP-7 into "AUC" |
| HP-7-mF2 | TAGGAGTAGCATGGGATCC**GTG**AGCGAGAATTACAGCAAGATTGTCGTTGGCACTGAT GGATCT | Mutating the "AUG" of HP-7 into "GUG" |
| HP-7-mF3 | TAGGAGTAGCATGGGATCC**AAC**AGCGAGAATTACAGCAAGATTGTCGTTGGCACTGAT GGATCT | Mutating the "AUG" of HP-7 into "AAC" |
| HP-7-mF4 | TAGGAGTAGCATGGGATCC**AAG**AGCGAGAATTACAGCAAGATTGTCGTTGGCACTGAT GGATCT | Mutating the "AUG" of HP-7 into "AAG" |
| HP-12-mF1 | TAGGAGTAGCATGGGATCC**ATC**GCTGTATACGAACTCCCAGAACTCGACTACGCATAC GACGCTCTCGA | Mutating the "AUG" of HP-12 into "AUC" |
| HP-12-mF2 | TAGGAGTAGCATGGGATCC**GTG**GCTGTATACGAACTCCCAGAACTCGACTACGCATAC GACGCTCTCGA | Mutating the "AUG" of HP-12 into "GUG" |

**Table S2.** continued.

| Primer | Sequence (5′-3′)[a] | Intension |
|---|---|---|
| HP-12-mF3 | TAGGAGTAGCATGGGATCC**AAC**GCTGTATACGAACTCCCAGAACTCGACTACGCATACGACGCTCTCGA | Mutating the "AUG" of HP-12 into "AAC" |
| HP-12-mF4 | TAGGAGTAGCATGGGATCC**AAG**GCTGTATACGAACTCCCAGAACTCGACTACGCATACGACGCTCTCGA | Mutating the "AUG" of HP-12 into "AAG" |
| SD-M1-R | TCCTCGCCCTTGCTCACCATCATTAGTTGTCC**TGG**TTTTGGTTGGATGCGTTCCATTCGCTCTGACCGTG | Mutating the "AGGAGG" of SD into "ACCAGG" |
| SD-M2-R | TCCTCGCCCTTGCTCACCATCATTAGTTG**TGGTGG**TTTTGGTTGGATGCGTTCCATTCGCTCTGACCGTG | Mutating the "AGGAGG" of SD into "ACCACC" |
| SD-M1-EM-R | AACAGCTCCTCGCCCTTGCTCAC**GAT**TAGTTGTCC**TGG**TTTTGGTTGGATGCGTTCCATTCGCTCTGACCGTG | Mutating the "AGGAGG" of SD into "ACCAGG" and the "AUG" of EGFP into "AUC" |
| SD-M2-EM-R | AACAGCTCCTCGCCCTTGCTCAC**GAT**TAGTTG**TGGTGG**TTTTGGTTGGATGCGTTCCATTCGCTCTGACCGTG | Mutating the "AGGAGG" of SD into "ACCACC" and the "AUG" of EGFP into "AUC" |
| AP | CGCGTCGACTAGTACGGGGGGGGGG | oligo anchor primer for 5′RACE |
| GSP1-EGFP | CCGGTGGTGCAGATGAACTT | gene-specific primer1 for EGFP |
| GSP2-EGFP | GCTGAACTTGTGGCCGTTTA | gene-specific primer2 for EGFP |
| GSP1-cg0124 | GAAGGGTTCGGTGCGGTCGA | gene-specific primer1 for cg0124 |
| GSP2-cg0124 | GGTAAACAACATCGGCGTAT | gene-specific primer2 for cg0124 |
| Library-F | GTAGGAGTAGCATGGGATCCATGNNNNNNNNNNNNNNNNNNNNNNNNNNNNNNNNNNNNNNNNNNNNNNNNNNNNNNNNNNNNNNNNNNAAAGGAGGACAACTAATG | Constructing the fore-cistron library |
| Library-R | CATGGATCCCATGCTACTCCTACCAACCAAGGTGCGACCACCCTGGGCCCGTTAATATTC | Constructing the fore-cistron library |
| NGS-F | TGGTGCCCTAAACGGGGGAATA | Amplifying the fore-cistron fragment for sequencing |
| NGS-R | GTCGCCGTCCAGCTCGACCAGGATG | Amplifying the fore-cistron fragment for sequencing |

[a] Red sequences represent homology arms, green sequences represent SD sequences. Underlined bold sequences represent mutation.

**Table S3. Identification of transcription initiation sites (TSPs) of leaderless BCDs.**

| Leaderless BCD[a] | Sequence (5′-3′)[b] |
| --- | --- |
| HP-1 | …GTAGGAGTAGCATGGGATCC**ATGGCAAACGTCAACATCAAGCCGCTTGAGGACAAGATCCTCGTTCAGATCAACGAAGCAGA** |
| HP-2 | …GTAGGAGTAGCATGGGATCC**ATGACTGAACGTACTCTCATCCTTATCAAGCCAGACGGTGTTACCAACGGACACGTCGGCGA** |
| HP-3 | …GTAGGAGTAGCATGGGATCC**ATGCCTATCGCAACTCCCGAGGTCTATAACGAGATGCTCGATCGTGCTAAGGAAGGCGGATT** |
| HP-4 | …GTAGGAGTAGCATGGGATCC**ATGGGGTCCATGGCTAAAACACATTTTCAAGGCAACGAAACTGCTACCTCCGGCGAACTGCC** |
| HP-5 | …GTAGGAGTAGCATGGGATCC**ATGAGCGAGAATTACAGCAAGATTGTCGTTGGCACTGATGGATCTAAGTCGTCCCTTCTAGC** |
| HP-6 | …GTAGGAGTAGCATGGGATCC**ATGGCCCGTGTAGTTGTCAATGTCATGCCTAAGGCTGAGATTCTGGATCCCCAGGGGCAGGC** |
| HP-7 | …GTAGGAGTAGCATGGGATCC**ATGACTAACGGAAAATTGATTCTTCTTCGTCACGGTCAGAGCGAATGGAACGCATCCAACCA** |
| HP-8 | …GTAGGAGTAGCATGGGATCC**ATGGCTGAAATCATGCACGTATTCGCTCGCGAAATTCTCGACTCCCGCGGTAACCCAACCGT** |
| HP-9 | …GTAGGAGTAGCATGGGATCC**ATGAGCGATATTCGTATGGCAGCCCAGGGTGGGCCTGGTTTCGGAAATGACGTCTTTGATCG** |
| HP-10 | …GTAGGAGTAGCATGGGATCC**ATGCGACTCGTACTCCTCGGACCTCCCGGTGCTGGTAAGGGCACCCAGGCTGCAATTCTCTC** |
| HP-11 | …GTAGGAGTAGCATGGGATCC**ATGATTGGAGCACCACCCGACATGGGCAATGTGTACAACAACATCACCGAAACCATCGGCCA** |
| HP-12 | …GTAGGAGTAGCATGGGATCC**ATGGCTGTATACGAACTCCCAGAACTCGACTACGCATACGACGCTCTCGAGCCACACATCGC** |

[a] HP-1 to HP-12 represented leaderless BCD containing different fore-cistron sequences. [b] 5´RACE determined TSPs (+1) were given in red letters. The underlined sequence represented the sequence of $P_{H36}$ and the bold sequence represented the fore-cistron sequence.

**Table S4. The fore-cistron sequence of 42 clones randomly selected from H-group.**

| Clone | The fore-cistron sequence (5′-3′) |
|---|---|
| 1 | ATGCTTTAGTCCCTATCGGGGGCTTTTATATCTGCTCCGTAATATATACCGGGGCCTCAGTT |
| 2 | ATGCTTGAGCCTGTCTGGTTCAGTATTTCTCACTACAACATTATCTCAGTAGGACTGATCAT |
| 3 | ATGAGACTACTTCTAAATTGTTTTGCGCCATTGTTCATACAATTTCTAAGCTATTTGCAATT |
| 4 | ATGACCTCTATAGTACAGAAGAGAAACTCTTTAAAGCCCATTTGGTTTCGCGTTTTGGTGTT |
| 5 | ATGCTTACATTCTTCGGTACTAAATCTACTGGACCCATCGAATCTTTGCCGATCGAACTGGA |
| 6 | ATGTATTTTTCTTGTCGTGATGTATTTTATTTCGTGTGTAATTTTCCTTTCCTTCTTGCCGG |
| 7 | ATGATGCGACCTTCTGTCCTAAGATTTCCAGGCCAGTGCGTTCTGGGAATAGGCCGTACTGT |
| 8 | ATGTTCGTTCTTAGAGTATGCTGTAATCAGTCCGGATGTACTAACACGAGAATAATTCTTAC |
| 9 | ATGATTCGTTCCCAATCTTTCTCCCTTGGATATTTTCTAAAACATTATTTTCTACTGTTTTT |
| 10 | ATGCGTTATTCCCGACCCTCGATTATTCATTATCGGAGTTGTTCTTGATCCCATCCAGCAAT |
| 11 | ATGGCAATCTTTCTGACTATATTTTCTGTGTTTCTTACTCTTTACTCAAATCTTTTTCTATT |
| 12 | ATGTGGAAAATCGTTTTTGGATCCATTTACATTCTTTTATTTTTCAATATGTACTGTAATAC |
| 13 | ATGAAGAGTGTATATAATTGTGAAAGCGTGCTTTTCTTTTCCTATATGAACATTTCCCCATT |
| 14 | ATGGTATTCCATTGTTTTCAAACGGATTTAGGTCTTTCTGGTCCAGGCCCCGGTTTACGTGT |
| 15 | ATGCTTTCAAAAGGATTTCACTTCAGAATTTTTGTTGTTACTGATATTTCTACCTCGTGTTC |
| 16 | ATGTTTACTTCCGGTAACTCTTATTGCGATTCAATGTTATCAAAGAATGCTTTATCCTTTAG |
| 17 | ATGATTTGTTTTTTATTATCTCAGTTTCTACTCGATGATGGCAGCTTGTTGGCTTGCGTTTC |
| 18 | ATGGTGTTGACCCATTGGGCGCGGTGCGTGTTCACGCGTCACGGACGGGTTTGATTCTTTTG |
| 19 | ATGATGCAATTAACAGTACCTTGGAGCAAATTGGTTAATATTTCTTTTCTGATTCTTCAATT |
| 20 | ATGAATTCCTGTTATCTGGATCTTTCTTGTTATGTGTATCTATTACCGTATGGTTTGAATGC |
| 21 | ATGATCTTTCTTCTAGGGTATCCAATTCGTTTACGACGCAAATCTTGGTTTTGTGTGAATCA |
| 22 | ATGAGCTGAACATCTCCGTGTGGAGTACGCTCATCGATCCTGGGGAGCGAATTCTTCATGCG |
| 23 | ATGACATAATCGTCGGGCTACGTATTTATTTAGCGTTTCCCTACGTCACGGTAGTATCTATT |
| 24 | ATGTCTCATAGCACTTTCCTGGTTGAGTTAATCGTGAATTTCATATTATGGTTCTGTGTAAC |
| 25 | ATGCGTGTTTCACTTCCGTCTGTGTCTCTGTTTTTTCTTAAGTTACAAGGTACACTTTCTCT |
| 26 | ATGATGTTATTTGTTAATTTAGATTCGTCTACAGTAAGTTGGGGCTGCTTTGTTTGTTATCC |
| 27 | ATGGCGTGTTGCCCTGTCTCGGCAATATAGCATAGGTTTCCTACCTCCTCTATTTACCCACC |
| 28 | ATGGACTTATATACCGTTATAGTCCACGTCACATGGGAAGTGATATAATGGCAAACAATACA |
| 29 | ATGTGTTTATTTCTGCTAGTGTAAACATGACGTACATAGACTATGAGAATTTCCTATATCTC |
| 30 | ATGCATGACCGAGTGATTCGTTTATTTCATCTATCTACTGCTCTGTTTCTATATTATGTTCA |
| 31 | ATGAAACTCGTGCGGCTTGATTCTAGCATGTGATACACTTCTTCATAATGTATTCTAGAATC |
| 32 | ATGATCTTGACAAATTACTTTTGCTCGCTTAGACTGAATCAACGAAGTAAATATATCACATC |
| 33 | ATGGTAGATATCCGATTTTTGCTACAAGTACGTAACCTATCTTCCTCGCAACTGACTTGTAT |
| 34 | ATGGATAAATTTCTGATTCGTGGCGTACCTTTTCATGTCGGTTGCTTTTCGATGGTCTCTCA |
| 35 | ATGTTTCCTGTTTTTTTGTACGGTTCAATTTCGCTGAGTACTTCTATCCGACCCAATGAGTT |
| 36 | ATGTCAATTTCGTGTAAGTGTGTCTATTCCAAATCCAAAATTGTTTCTGGGGTTCCCGTTAC |
| 37 | ATGATTCATCTCTGTTTGGTCTTTTATTTTATTTCACAGTCTTATACGTTTTTGCCTTTTGT |
| 38 | ATGCCCGTTCCATTTTTCGTTTACATTGATTTTCCAATTGTTTGTTTATGGTTACTTTCTAT |
| 39 | ATGTTACGTGTTAAATCTGGAGCATCTAAGTCACTTTTTTGCTTTCGTCTGACCCACAACTT |
| 40 | ATGACATCTCATTTTTTTATAAGCTCCTTCCCGTCAGGTCTTAGTATTTTGTTAATGCTTGT |
| 41 | ATGGGAGCAATTAGTTTGTTTTTTTCGTTTTGTTTTAATACACCCGTATATCATATATTCTT |
| 42 | ATGTAGCGATCGCCACTGGACGATATCTGTATACTTTGATCAACATTCCGTTCTTATGTAGT |
